# Mapping phenotypic and aetiological associations between ADHD and physical conditions in adulthood in Sweden: a genetically informed register study

**DOI:** 10.1016/S2215-0366(21)00171-1

**Published:** 2021-09

**Authors:** Ebba Du Rietz, Isabell Brikell, Agnieszka Butwicka, Marica Leone, Zheng Chang, Samuele Cortese, Brian M D'Onofrio, Catharina A Hartman, Paul Lichtenstein, Stephen V Faraone, Ralf Kuja-Halkola, Henrik Larsson

**Affiliations:** a Department of Medical Epidemiology and Biostatistics, Karolinska Institutet, Stockholm, Sweden; b Child and Adolescent Psychiatry Stockholm, Stockholm Health Care Services, Stockholm, Sweden; c Department of Child Psychiatry, Medical University of Warsaw, Warsaw, Poland; d Janssen-Cilag, Solna, Sweden; e Centre for Innovation in Mental Health, School of Psychology, Life and Environmental Sciences, University of Southampton, Southampton, UK; f Clinical and Experimental Sciences (CNS and Psychiatry), Faculty of Medicine, University of Southampton, Southampton, UK; g Division of Psychiatry and Applied Psychology, School of Medicine, University of Nottingham, Nottingham, UK; h National Institute of Health Research (NIHR) Nottingham, Biomedical Research Centre, Nottingham, UK; i Department of Child and Adolescent Psychiatry, NYU Grossman School of Medicine, New York City, NY, USA; j Department of Psychological and Brain Sciences, Indiana University, Bloomington, IN, USA; k Department of Psychiatry, University of Groningen, University Medical Center Groningen, Interdisciplinary Center Psychopathology and Emotion regulation (ICPE), Groningen, Netherlands; l Department of Psychiatry and Department of Neuroscience and Physiology, SUNY-Upstate Medical University, Syracuse, NY, USA; m School of Medical Sciences, Örebro University, Örebro, Sweden

## Abstract

**Background:**

Emerging evidence suggests increased risk of several physical health conditions in people with ADHD. Only a few physical conditions have been thoroughly studied in relation to ADHD, and there is little knowledge on associations in older adults in particular. We aimed to investigate the phenotypic and aetiological associations between ADHD and a wide range of physical health conditions across adulthood.

**Methods:**

We did a register study in Sweden and identified full-sibling and maternal half-sibling pairs born between Jan 1, 1932, and Dec 31, 1995, through the Population and Multi-Generation Registers. We excluded individuals who died or emigrated before Jan 1, 2005, and included full-siblings who were not twins and did not have half-siblings. ICD diagnoses were obtained from the National Patient Register. We extracted ICD diagnoses for physical conditions, when participants were aged 18 years or older, from inpatient (recorded 1973–2013) and outpatient (recorded 2001–13) services. Diagnoses were regarded as lifetime presence or absence. Logistic regression models were used to estimate the associations between ADHD (exposure) and 35 physical conditions (outcomes) in individuals and across sibling pairs. Quantitative genetic modelling was used to estimate the extent to which genetic and environmental factors accounted for the associations with ADHD.

**Findings:**

4 789 799 individuals were identified (2 449 146 [51%] men and 2 340 653 [49%] women), who formed 4 288 451 unique sibling pairs (3 819 207 full-sibling pairs and 469 244 maternal half-sibling pairs) and 1 841 303 family clusters (siblings, parents, cousins, spouses). The mean age at end of follow-up was 47 years (range 18–81; mean birth year 1966); ethnicity data were not available. Adults with ADHD had increased risk for most physical conditions (34 [97%] of 35) compared with adults without ADHD; the strongest associations were with nervous system disorders (eg, sleep disorders, epilepsy, dementia; odds ratios [ORs] 1·50–4·62) and respiratory diseases (eg, asthma, chronic obstructive pulmonary disease; ORs 2·42–3·24). Sex-stratified analyses showed similar patterns of results in men and women. Stronger cross-disorder associations were found between full-siblings than between half-siblings for nervous system, respiratory, musculoskeletal, and metabolic diseases (p<0·007). Quantitative genetic modelling showed that these associations were largely explained by shared genetic factors (60–69% of correlations), except for associations with nervous system disorders, which were mainly explained by non-shared environmental factors.

**Interpretation:**

This mapping of aetiological sources of cross-disorder overlap can guide future research aiming to identify specific mechanisms contributing to risk of physical conditions in people with ADHD, which could ultimately inform preventive and lifestyle intervention efforts. Our findings highlight the importance of assessing the presence of physical conditions in patients with ADHD.

**Funding:**

Swedish Research Council; Swedish Brain Foundation; Swedish Research Council for Health, Working Life, and Welfare; Stockholm County Council; StratNeuro; EU Horizon 2020 research and innovation programme; National Institute of Mental Health.

## Introduction

ADHD is a neurodevelopmental disorder with an estimated worldwide prevalence of 3–7% in childhood and adolescence,^1^, ^2^ and around 3% in adulthood.^3^, ^4^ Twin and family studies have shown that ADHD has a high heritability, of around 70–80%,^5^ and molecular genetic studies have estimated that common genetic variants account for almost one-third of this heritability.^6^ Although there is a plethora of research that has shown the frequent co-occurrence of psychiatric disorders with ADHD,^7^ associations with physical health conditions have been less studied, despite health-care costs in ADHD being substantially driven by both psychiatric and physical comorbid conditions.^8^, ^9^ Physical conditions that have shown robust and replicated association with ADHD, especially in childhood, include metabolic disorders, sleep disorders, and asthma.^10^ A few studies have suggested tentative associations between ADHD and dementia,^11^ diseases of the basal ganglia and cerebellum (eg, Parkinson's disease),^12^ and cardiovascular disease.^13^ However, some studies have shown no association,^9^, ^10^ and several studies have been limited by small samples and retrospective reports. There is a scarcity of studies in older adults and studies with lifespan follow-up. Consequently, there is little detailed knowledge on physical diseases in ADHD beyond young-to-middle adulthood.

**Research in context**

**Evidence before this study**

We searched Medline for systematic reviews published, with no language restrictions, between database inception and Jan 1, 2021, on the association between adult ADHD and physical health conditions, using the search terms “systematic review”, “ADHD” or “attention deficit hyperactivity disorder”, “adult”, and “physical disease” or “somatic disease”. We identified the most recent systematic review on the topic (by Instanes and colleagues, published in 2018, including 126 studies published up to January, 2015) and updated it with an additional 19 studies retrieved using the same search strategy and syntax in the Medline database. The available body of research shows that only a few physical conditions have been thoroughly studied in relation to adult ADHD. Emerging evidence, however, suggests increased risk of several physical conditions in people with ADHD. Physical conditions that have shown robust and replicated association with ADHD include obesity, sleep disorders, and asthma, mostly in children. A small number of studies have suggested tentative associations between ADHD and age-related diseases. However, findings have been mixed as not all studies have shown an association, and several studies have been limited by small samples and retrospective reports. Consequently, there is little detailed knowledge on physical diseases in ADHD beyond young-to-middle adulthood.

**Added value of this study**

To our knowledge, this is the first large-scale register-based study of phenotypic and aetiological associations between ADHD and a broad range of physical health conditions across adulthood. We found that adults with ADHD were at increased risk of numerous physical conditions, across nervous system, respiratory, musculoskeletal, metabolic, circulatory, gastrointestinal, genitourinary, and skin disease groups, compared with adults without ADHD. Although there is little research on the associations between ADHD and age-related diseases, due to the scarcity of lifespan data sources, we showed that ADHD is linked to increased risk of cardiovascular disease, Parkinson's disease, and dementia. Quantitative genetic modelling showed that numerous associations were largely explained by shared genetic factors, with the exception of associations with nervous system and age-related diseases. These findings provide aetiological insights into the co-occurrence of ADHD and physical diseases.

**Implications of all the available evidence**

Detailed management guidelines for adults with ADHD and co-occurring physical diseases are scarce. However, emerging research findings have shown an increased risk of physical diseases in adult patients with ADHD, which highlights the importance of assessing the presence of physical conditions in these patients. Identifying co-occurring physical conditions could have important implications for treating adults with ADHD and for benefiting the long-term health and quality of life of patients. Emerging aetiological research findings can also be used to guide future research aiming to identify specific mechanisms contributing to risk of physical conditions in people with ADHD, which could ultimately inform preventive and lifestyle intervention efforts.

A register study in Denmark published in 2020 showed that behavioural disorders (including ADHD, conduct disorders, and childhood-emotional disorders) were significantly associated with a range of subsequent physical conditions. Associations with physical conditions were generally of similar or greater magnitude than associations between physical conditions and other psychiatric disorders.^14^ However, the extent to which these associations were specifically driven by ADHD was not investigated.^14^ A register study in Sweden published in 2020 showed that adults with ADHD were more likely to receive medications for a range of physical conditions, than adults without ADHD.^15^ Thus, epidemiological research suggests that adults with ADHD have an increased risk of a wide range of physical conditions; however, these have not yet been systematically mapped out.

Only a few studies have investigated whether aetiological factors underlying ADHD are shared with physical conditions. The few available quantitative and molecular genetic studies have revealed weak-to-moderate genetic associations between ADHD and conditions such as obesity, diabetes, asthma, epilepsy, and migraines.^6^, ^16^, ^17^, ^18^ Further research, using statistically high-powered methods and a broader range of diseases across adulthood, is needed to understand the aetiological processes underlying the increased occurrence of physical conditions in people with ADHD.

Higher risk of physical conditions in siblings of individuals with ADHD indicates that familial factors shared among siblings contribute to the co-occurrence of disorders. Differences in the magnitude of association across full-siblings and maternal half-siblings can point to the source of familial coaggregation (genetic or environmental). Full-siblings share on average 50% of their segregating genes, whereas half-siblings share 25%. Full-siblings and maternal half-siblings tend to share environmental factors, as they are often reared in the same household. Thus, a greater association in full-siblings than in half-siblings suggests that shared genetic factors contribute to the association between conditions.^18^

We aimed to investigate the phenotypic and aetiological associations between ADHD and a wide range of physical health conditions, and to examine the extent to which any observed associations are primarily genetic or environmental in origin, by estimating individual associations, familial coaggregation, and genetic and environmental contributions to the associations.

## Methods

### Study design and participants

We did a register study in Sweden and identified full-sibling and maternal half-sibling pairs born between Jan 1, 1932, and Dec 31, 1995, through the Population and Multi-Generation Registers.^19^ We excluded individuals who died or emigrated before Jan 1, 2005, and included full-siblings who were not twins and did not have half-siblings. Using personal identification numbers, we linked nation-wide registers. Data on ethnicity were not available in the registers. This study uses population national register data, which means that no individual patients were involved in the formulation of research questions, outcome measures, study design, implementation of the study, or dissemination of the research findings. The use of Swedish register data does not require informed consent. This study was approved by the Regional Ethical Review Board in Stockholm, Sweden (Dnr2013/862–31/5).

### Procedures

ICD diagnoses were obtained from the National Patient Register,^20^ which includes psychiatric inpatient admissions since 1973 and visits to outpatient specialist clinics since 2001, classified according to ICD-8 (1969–1986), ICD-9 (1987–1996), or ICD-10 (1997–present). We extracted ICD diagnoses for physical conditions, that occurred when participants were aged 18 years or older, from inpatient (recorded 1973–2013) and outpatient (recorded 2001–13) services. Diagnoses were treated as lifetime presence or absence. Although coverage of inpatient visits in the National Patient Register was incomplete until the early 1980s, it now has complete coverage since 1987, and currently more than 99% of all psychiatric and somatic discharges are registered.^21^ When outpatient visits were first recorded in 2001, the register covered 70–85% of diagnoses. The coverage quickly improved over time, and now covers 97% of all outpatient visits.^22^ Previous research has shown high positive predictive values for most inpatient register diagnoses when compared with information from medical records.^21^ People with ADHD were identified as individuals who had received an ADHD diagnosis (ICD-9 or ICD-10: 314/F90) or ADHD medication prescription (Anatomical Therapeutic Chemical [ATC] classification: N06BA01/N06BA02/N06BA04/N06BA09/N06BA12), or both, at age 18 years or older (recorded in the National Patient Register and Prescribed Drug Register^23^). Previous research has indicated high specificity for this register-based ADHD definition in Sweden,^24^ and shown that patterns of aetiological influences remain similar whether people with ADHD are identified through diagnoses or prescriptions.^25^ Only physicians specialised in psychiatry or neurology and responsible for ADHD treatment are authorised to prescribe the medication in Sweden, which supports that prescription of ADHD medication is a valid indicator of ADHD diagnoses.^26^

We based our data on an existing register linkage; we identified 35 relevant common physical conditions, and these were categorised into eight broader disease groups on the basis of ICD-10 structure (appendix pp 2–3). Analyses were run both for single conditions and broader disease groups (presence or absence of any diagnosis within the group). We excluded diagnoses of narcolepsy in the group of sleep disorders, due to the occasional off-label use of ADHD medication for narcolepsy.^27^

### Statistical analysis

For the co-occurrence and familial coaggregation analysis, we used logistic regression to estimate the associations between ADHD and physical conditions, by comparing the risk of physical conditions (outcomes) in individuals with and without ADHD (exposure). We fitted logistic regression models in full-siblings and maternal half-siblings separately, to examine the associations between ADHD in individuals (exposure individuals) and physical conditions in their siblings (outcome individuals). Associations are presented as odds ratios (ORs) with 95% CIs. Statistical comparisons of the ORs in full-sibling and maternal half-sibling cohorts were made, and significance was determined based on a false discovery rate-corrected p value at a threshold set at p=0·007.

In sensitivity analyses, we examined whether the identified familial associations between ADHD and physical conditions could be explained by direct effects of one condition on the other. This was examined in the full-sibling cohort, by adjusting for ADHD in the outcome individuals. If ORs remained significant after adjustment, the contribution of common familial risk factors to ADHD and the physical condition would be further supported. Detailed description of this rationale has been provided elsewhere.^28^ We stratified analyses by sex of exposure individuals to explore differences in men and women. Furthermore, we re-ran analyses excluding sibling pairs who were born more than 10 years apart, to ensure that the assumption of shared environment would be accurate despite the inclusion of sibling pairs with greater age differences.

Analyses included sex and birth year of both relatives as covariates to adjust for different follow-up lengths and cohort effects. Data management was done using SAS version 9.4, and analyses were run using Stata version 16.0. A cluster robust sandwich estimator was used to remove distributional assumptions and correct CIs for dependence of family data.

Quantitative genetic modelling was used to estimate the contribution of genetic and environmental factors to the associations between ADHD and physical conditions. We selected conditions that showed significantly stronger associations with ADHD across full-siblings than maternal half-siblings, as this suggests genetic factors contribute to the cross-disorder covariance. The liability-threshold model was used, where an underlying normally distributed liability of disease is assumed.^29^ If an individual has a diagnosis, the risk-liability is assumed to be higher than an estimated threshold. The inferred correlation between liabilities is equivalent to a tetrachoric correlation.^29^ We specified additive genetic, shared environmental, and non-shared environmental latent factors as sources of variance and covariance between ADHD and physical conditions.^30^ Genetic factors were fixed to correlate between siblings at their expected average sharing of co-segregating genes (full-siblings 50%; half-siblings 25%). Shared environmental factors (non-genetic components making siblings similar) were fixed to correlate at unity across full-siblings and half-siblings. Thus, we assumed the shared environmental factors to be equally shared across full-siblings and maternal half-siblings, as previous literature has shown strong support for this assumption in Swedish registers.^30^ Non-shared environmental factors were fixed to correlate at 0 across all siblings, measuring non-genetic components making siblings dissimilar.

Analyses included sex and birth year as covariates, and models were fitted using the weighted least-squares method in OpenMx version 2.15.5. Because half-siblings tend to display higher rates of disorders than full-siblings,^31^, ^32^ prevalence was allowed to vary across full-siblings and half-siblings. To obtain valid SEs when including multiple sibling pairs per family, we used non-parametric bootstrap sampling.^33^ A bootstrap sample was created by repeatedly drawing families from 1 841 303 family clusters. For a given bootstrap sample, the estimates from the quantitative genetic model were computed. After repeating this process across 1000 bootstrap samples, we computed 95% CIs as the 2·5 and 97·5 percentiles of bootstrap replicates for parameters (allowing skewed CIs).

### Role of the funding source

The funders of the study had no role in study design, data collection, data analysis, data interpretation, or writing of the report.

## Results

4 789 799 individuals were identified (4 176 415 full-siblings and 613 384 maternal half-siblings; 688 937 individuals died or emigrated before 2005 and were excluded), who formed 4 288 451 unique sibling pairs (3 819 207 full-sibling pairs and 469 244 maternal half-sibling pairs) and 1 841 303 family clusters (siblings, parents, cousins, spouses).

The full cohort included 2 449 146 (51%) men and 2 340 653 (49%) women, and the mean age at end of follow-up was 47 years (range 18–81; table 1). Lifetime prevalences for all conditions are shown in table 2 (appendix pp 4–9). There was an overall trend of higher prevalence of ADHD, and other conditions, in half-siblings than full-siblings, in line with previous studies.^31^, ^32^

**Table 1:** Participant characteristics

		**Participants (n=4 789 799)**
Birth year		
	1932–44	585 669 (12%)
	1945–57	1 041 909 (22%)
	1958–70	1 098 306 (23%)
	1971–83	1 029 784 (21%)
	1984–95	1 034 131 (22%)
Age difference, years (mean [SD])		5·84 (4·04)
Sex		
	Male	2 449 146 (51%)
	Female	2 340 653 (49%)
Full-siblings		
	Individuals	4 176 415 (87%)
	Unique pairs	3 819 207
	Analytical pairs	7 638 414
Maternal half-siblings		
	Individuals	613 384 (13%)
	Unique pairs	469 244
	Analytical pairs	938 488

Data are n or n (%) unless otherwise stated. Age difference is the difference in years of age between siblings in a pair. Analytical pairs are the number of sibling pairs used in between-sibling association analyses.

**Table 2:** Lifetime prevalence of ADHD and physical conditions in the full cohort

		**Participants (n=4 789 799)**
ADHD		61 960 (1·29%)
Circulatory system		
	Hypertension	462 113 (9·65%)
	Ischaemic heart disease	184 737 (3·86%)
	Pulmonary disease	38 117 (0·80%)
	Atrial fibrillation	168 986 (3·53%)
	Heart failure	71 071 (1·48%)
	Stroke	97 390 (2·03%)
	Peripheral vascular disease	62 186 (1·30%)
Endocrine or metabolic		
	Type 1 diabetes	80 741 (1·69%)
	Type 2 diabetes	154 209 (3·22%)
	Thyroid disorders	149 861 (3·13%)
	Obesity	113 768 (2·38%)
	Gout	20 686 (0·43%)
Gastrointestinal		
	Coeliac disease	18 952 (0·40%)
	Ulcer or chronic gastritis	73 216 (1·53%)
	Acute appendicitis	154 572 (3·23%)
	Fatty liver disease	5825 (0·12%)
	Alcohol-related liver disease	10 348 (0·22%)
	Inflammatory bowel disease	64 790 (1·35%)
	Gallstone disease	213 531 (4·46%)
Genitourinary		
	Glomerular disease	22 966 (0·48%)
	Urolithiasis	128 582 (2·68%)
	Kidney infections	61 295 (1·28%)
Musculoskeletal		
	Rheumatoid arthritis	42 985 (0·90%)
	Arthrosis	322 417 (6·73%)
	Connective tissue disease	68 865 (1·44%)
	Dorsalgia (back pain)	298 076 (6·22%)
Nervous system		
	Parkinson's disease	12 569 (0·26%)
	Dementia	20 729 (0·43%)
	Epilepsy	62 142 (1·30%)
	Migraine	70 426 (1·47%)
	Sleep disorders	122 980 (2·57%)
Respiratory		
	Asthma	143 828 (3·00%)
	COPD	74 868 (1·56%)
Skin		
	Eczema	50 057 (1·05%)
	Psoriasis	89 639 (1·87%)

Data are n (%). COPD=chronic obstructive pulmonary disease.

Individuals with ADHD had significantly increased risk of all physical conditions except rheumatoid arthritis, after adjusting for sex and birth year, compared with individuals without ADHD (p values <0·007; table 3). The strongest associations were with alcohol-related liver disease (OR 4·70 [95% CI 3·96–5·58]), sleep disorders (4·62 [4·43–4·82]), chronic obstructive pulmonary disease (COPD; 3·24 [2·96–3·56]), epilepsy (2·99 [2·82–3·16]), fatty liver disease (2·94 [2·38–3·63]), and obesity (2·67 [2·55–2·78]). Full-siblings of individuals with ADHD had significantly increased risk for most physical conditions; these associations were generally attenuated in maternal half-siblings (table 3). The associations between full-siblings were significantly stronger than between maternal half-siblings for type 1 diabetes, obesity, kidney infections, dorsalgia, migraine, sleep disorders, asthma, and COPD (p values <0·007).

**Table 3:** Associations between ADHD and physical conditions in individuals and between full-siblings and maternal half-siblings

	**OR (95% CI)**			p value*
	Within individuals	Full-siblings	Maternal half-siblings	
**Circulatory system**				
Hypertension	1·65 (1·57–1·74)	1·10 (1·05–1·15)	1·04 (0·96–1·29)	0·26
Ischaemic heart disease	1·34 (1·21–1·49)	1·16 (1·08–1·26)	1·12 (0·97–1·29)	0·62
Pulmonary disease	1·90 (1·66–2·18)	1·05 (0·92–1·20)	1·07 (0·86–1·32)	0·91
Atrial fibrillation	1·32 (1·22–1·43)	1·05 (0·98–1·13)	1·06 (0·95–1·19)	0·90
Heart failure	1·69 (1·48–1·94)	1·16 (1·02–1·30)	1·18 (0·97–1·45)	0·84
Stroke	1·90 (1·72–2·10)	1·19 (1·09–1·30)	1·05 (0·90–1·23)	0·17
Peripheral vascular disease	1·78 (1·57–2·02)	1·14 (1·02–1·27)	1·12 (0·93–1·35)	0·88
**Endocrine or metabolic**				
Type 1 diabetes	1·52 (1·40–1·64)	1·15 (1·07–1·24)	0·92 (0·81–1·04)	0·0023
Type 2 diabetes	2·01 (1·87–2·17)	1·21 (1·13–1·30)	1·04 (0·93–1·17)	0·030
Thyroid disorders	1·72 (1·62–1·82)	1·18 (1·12–1·25)	1·12 (1·03–1·22)	0·28
Obesity	2·67 (2·55–2·78)	1·61 (1·53–1·69)	1·26 (1·18–1·34)	<0·0001
Gout	1·78 (1·48–2·15)	1·31 (1·10–1·54)	1·08 (0·81–1·43)	0·26
**Gastrointestinal**				
Coeliac disease	1·38 (1·21–1·57)	1·07 (0·95–1·22)	0·95 (0·77–1·16)	0·29
Ulcer or chronic gastritis	2·45 (2·26–2·66)	1·31 (1·21–1·42)	1·33 (1·16–1·51)	0·88
Acute appendicitis	1·24 (1·17–1·31)	1·07 (1·02–1·12)	1·06 (0·99–1·14)	0·95
Fatty liver disease	2·94 (2·38–3·63)	1·36 (1·06–1·74)	1·01 (0·65–1·56)	0·24
Alcohol-related liver disease	4·70 (3·96–5·58)	1·90 (1·54–2·34)	1·49 (1·11–1·99)	0·18
Inflammatory bowel disease	1·23 (1·13–1·34)	1·05 (0·97–1·13)	1·06 (0·94–1·18)	0·90
Gallstone disease	1·77 (1·69–1·86)	1·22 (1·16–1·28)	1·15 (1·07–1·23)	0·15
**Genitourinary**				
Glomerular disease	2·02 (1·78–2·30)	1·29 (1·14–1·45)	1·21 (1·01–1·46)	0·25
Urolithiasis	1·54 (1·45–1·64)	1·14 (1·07–1·21)	1·09 (1·00–1·20)	0·46
Kidney infections	2·30 (2·15–2·47)	1·40 (1·28–1·47)	1·13 (1·01–1·25)	0·0023
**Musculoskeletal**				
Rheumatoid arthritis	1·08 (0·92–1·25)	1·02 (0·91–1·14)	1·08 (0·91–1·29)	0·59
Arthrosis	1·31 (1·23–1·38)	1·11 (1·06–1·17)	0·99 (0·92–1·08)	0·019
Connective tissue disease	1·75 (1·60–1·93)	1·16 (1·07–1·27)	1·09 (0·95–1·25)	0·44
Dorsalgia (back pain)	2·39 (2·32–2·47)	1·40 (1·36–1·45)	1·23 (1·17–1·29)	<0·0001
**Nervous system**				
Parkinson's disease	1·50 (1·08–2·09)	1·04 (0·78–1·39)	1·66 (1·01–2·73)	0·11
Dementia	2·44 (1·86–3·19)	1·08 (0·79–1·29)	1·35 (0·83–2·21)	0·30
Epilepsy	2·99 (2·82–3·16)	1·40 (1·31–1·50)	1·21 (1·09–1·34)	0·019
Migraine	1·97 (1·86–2·09)	1·37 (1·29–1·46)	1·13 (1·03–1·23)	0·0003
Sleep disorders	4·62 (4·43–4·82)	1·58 (1·50–1·67)	1·33 (1·22–1·44)	0·0005
**Respiratory**				
Asthma	2·42 (2·33–2·52)	1·41 (1·35–1·48)	1·22 (1·14–1·30)	0·0002
COPD	3·24 (2·96–3·56)	1·69 (1·54–1·84)	1·33 (1·14–1·54)	0·0070
**Skin**				
Eczema	1·42 (1·32–1·53)	1·03 (0·96–1·11)	1·05 (0·94–1·17)	0·82
Psoriasis	1·39 (1·29–1·49)	1·22 (1·15–1·31)	1·16 (1·06–1·28)	0·77

Analyses are adjusted for sex and birth year. False discovery rate-corrected p-value threshold for significance set at 0·007. COPD=chronic obstructive pulmonary disease. OR=odds ratio.

* p values are for comparison of ORs in full-siblings versus in maternal half-siblings.

**Figure 1:**
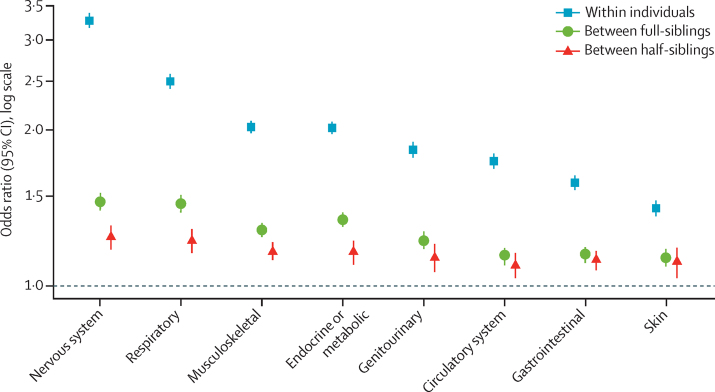
Associations between ADHD and broad disease groups in individuals and between full-siblings and maternal half-siblings

In the analyses of the eight broader disease groups, the strongest associations with ADHD were with nervous system (OR 3·27 [95% CI 3·17–3·37]), respiratory (2·49 [2·40–2·59]), musculoskeletal (2·03 [1·97–2·09]), and metabolic (2·02 [1·96–2·09]) diseases. The associations between ADHD and these four disease groups were significantly stronger between full-siblings than between maternal half-siblings (p values <0·007; figure 1; appendix p 10).

When adjusting for ADHD in outcome individuals, in the full-sibling cohort, we found that, even though ORs were slightly attenuated, the significant associations identified between ADHD and physical conditions across full-siblings mostly remained (appendix p 11). Only for peripheral vascular disease and fatty liver disease were associations with ADHD no longer significant across full-siblings, although CIs overlapped with the original estimates. When we re-ran analyses excluding sibling pairs who were born more than 10 years apart, we found that the overall pattern of results remained although CIs became wider, especially for associations in half-sibling pairs (appendix p 12). ORs for type 1 diabetes, kidney infections, and COPD were no longer significantly different between full-siblings and half-siblings.

Sex-stratified analyses revealed similar patterns of results in men and women, although CIs were wider in the stratified analyses compared with the original estimates, due to lower statistical power (appendix pp 13–14). Sex-stratified associations showed that the increased risk of atrial fibrillation, urolithiasis, sleep disorders, and asthma in women with ADHD was significantly higher than in men with ADHD (non-overlapping CIs), whereas the increased risk of thyroid disorder in women with ADHD was lower than in men with ADHD.

**Figure 2:**
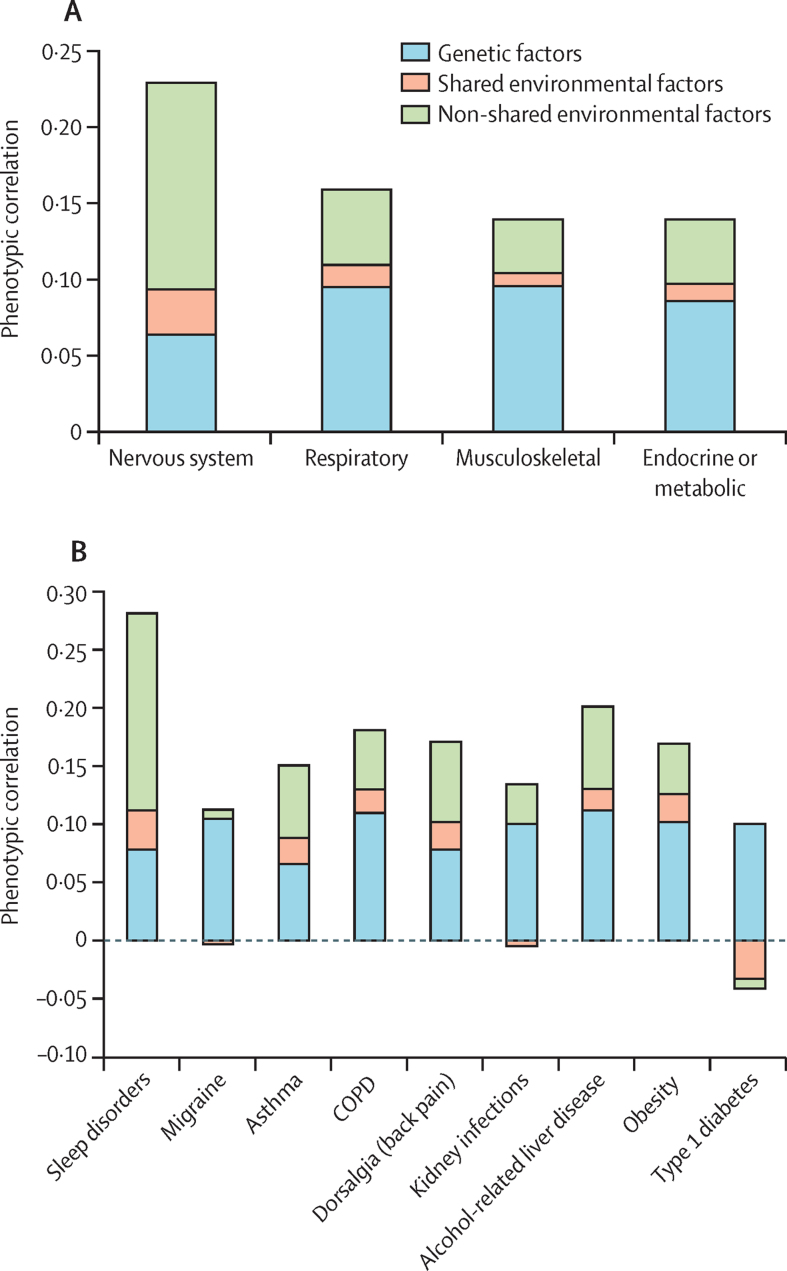
Contributions of additive genetic factors, shared environmental factors, and non-shared environmental factors on phenotypic correlations Contributions of additive genetic factors, shared environmental factors, and non-shared environmental factors on phenotypic correlations between ADHD and broader disease groups (A) and physical conditions (B). Quantitative genetic modelling was done for selected physical conditions that showed significantly stronger associations with ADHD across full-siblings than across maternal half-siblings. The y-axis is the tetrachoric correlation with ADHD. Negative estimates reflect that the aetiological factors have negative contributions to the phenotypic correlation. COPD=chronic obstructive pulmonary disease.

In the quantitative genetic analyses, the strongest phenotypic correlation was observed between ADHD and nervous system disorders (0·23 [95% CI 0·23–0·24]; appendix p 16); genetic factors explained 28% (95% CI 7–49), shared environmental factors explained 13% (3–22), and non-shared environmental factors explained 59% (47–71) of the correlation (figure 2). When examining nervous system disorders separately, this pattern of aetiological influences was seen for sleep disorders, whereas the correlation between ADHD and migraine was almost completely explained by genetic factors. Significant phenotypic correlations were also observed between ADHD and metabolic, respiratory, and musculoskeletal disease groups (0·14–0·16); genetic factors explained 60–69% of correlations and the remainder was explained by non-shared environmental factors (figure 2; appendix pp 15–17).

## Discussion

In this large-scale, register-based study we showed that adults with ADHD are at increased risk of a wide range of physical health conditions, compared with adults without ADHD. ADHD was associated with 34 (97%) of 35 physical conditions studied, and associations were strongest with nervous system disorders (particularly sleep disorders), and respiratory, musculoskeletal, and metabolic diseases. Similar patterns of associations with physical disorders were found in men and women. Several conditions within these disease groups have been linked with ADHD previously, often in smaller clinical studies. There is little research on associations between ADHD and age-related diseases, due to the scarcity of lifespan data sources, but we showed that ADHD is linked with increased risk of cardiovascular disease, Parkinson's disease, and dementia (1·32–2·44 times increased odds).

Between-sibling analyses showed that full-siblings of individuals with ADHD were at increased risk for numerous physical conditions, suggesting that shared familial factors contributed to the co-occurrence of the conditions. This inference was further supported by sensitivity analyses where we found that most significant associations remained after adjusting for ADHD in the sibling of the exposure individual. If these physical conditions were simply a consequence of behaviours driven by ADHD (eg, impulsive eating, taking ADHD medication) we would expect the associations to disappear after adjusting for ADHD in the sibling. However, as the associations remained significant, this finding supports the idea that ADHD and these physical conditions share aetiological components.

Significantly stronger associations with ADHD were found between full-siblings than between half-siblings for nervous system, respiratory, musculoskeletal, and metabolic disease groups, suggesting that the shared familial liability is partly due to genetics. Quantitative genetic modelling showed that these associations were largely explained by shared genetic factors. These findings are consistent with reports of genetic correlations between ADHD and obesity and diabetes from genome-wide association studies.^6^ It is possible that some of the identified genetic associations, such as between ADHD and respiratory disease and liver disease, reflect indirect genetic effects, mediated by risk factors such as obesity, smoking, and alcohol abuse. These are established risk factors for physical diseases, and have shown genetic associations with ADHD.^6^ Furthermore, ADHD is frequently comorbid, and shares a strong genetic basis with other psychiatric disorders.^6^, ^30^ Thus, associations between ADHD and physical conditions might be partly explained by genetic factors shared with other psychiatric issues, which would be important to investigate in future studies. The association between ADHD and nervous system diseases, however, was to a larger extent explained by non-shared environmental factors. These factors could reflect: first, causal environmental risk factors that influence both ADHD and nervous system diseases; or second, that one disorder increases the risk of the other disorder via environmental mechanisms; or both. These findings highlight the potential for identifying modifiable factors to alleviate nervous system diseases in adults with ADHD. An exception was migraine, which showed stronger (almost complete) genetic overlap with ADHD, in line with previous evidence from a genome-wide association study.^34^

It is also important to highlight associations that showed no evidence of shared familial liability. Between-sibling associations were not significant between ADHD and Parkinson's disease, dementia, inflammatory bowel disease, coeliac disease, rheumatoid arthritis, eczema, pulmonary disease, or atrial fibrillation. One possible explanation for no association with these conditions is limited statistical power due to low cross-disorder concordances. These are especially low for late-onset diseases, by contrast with ADHD which has childhood-onset and has only recently been commonly diagnosed in Sweden,^35^ and especially in half-siblings, as the proportion of half-siblings relative to full-siblings was lower in older generations. Other explanations might be that the higher risk for these physical conditions in ADHD is due to common causal environmental risk factors, or that one condition increases the risk of the other via environmental mechanisms, or both.

Our findings can guide future research on physical conditions in patients with ADHD. Whereas the co-occurrence of ADHD and several physical conditions implicated shared genetic factors, other conditions showed evidence that suggests unique environmental or directional effects. These findings highlight important directions for future research on more targeted biological mechanisms and modifiable risk factors, which could be informative for diagnosis, prevention, and treatment of physical conditions in patients with ADHD.^10^ Our findings also have more immediate clinical implications, highlighting that it is important for clinicians to assess the presence of physical conditions in adults with ADHD and consider the long-term associations of ADHD. Detailed treatment guidelines for adults with ADHD and co-occurring physical disease are largely absent. Identifying co-occurring physical conditions could have important implications for treating ADHD, as stimulant therapy requires careful monitoring in patients with cardiac disease, hypertension, and liver failure,^36^ whereas it might have positive effects for other conditions such as obesity and sleep disorders.^10^ ADHD might also impact the outcomes of physical conditions. For example, a German registry-study found that patients with ADHD and type 1 diabetes had poor metabolic control compared with patients with diabetes but without ADHD.^37^

This is, to our knowledge, the first large-scale study using a representative population cohort to examine phenotypic and aetiological associations between ADHD and a wide range of clinically assessed physical conditions across adulthood. It is important, however, to interpret results in the context of the limitations of the study. We relied on register-based diagnoses, and therefore findings rely on diagnosed patients who are potentially more severely affected than individuals who do not receive or seek health-care support. Furthermore, we did not directly test the effect of ADHD medication on physical conditions, as information on medication was not available before 2005. However, ADHD medications were not licensed for treatment in adults in Sweden before 2008,^38^ which suggests that the adults in this study, and in particular older adults, were unlikely to have been prescribed ADHD medications for long periods of time before the outcome.

During the follow-up period, there were changes in diagnostic practices for ADHD (eg, rate of ADHD diagnoses increased five-fold between 2004 and 2015)^35^ and, as noted, the coverage of the patient register has improved over time. Incomplete coverage by the register might introduce outcome misclassification bias (ie, false negatives), which would be most likely to bias estimates towards the null. Thus, our findings might reflect conservative estimates of true associations. The Multi-Generation Register contains available information on 98% of mothers and 95% of fathers of individuals.^19^, ^39^ Individuals with incomplete parental information are mainly those born before 1947, and those with parents who were born in another country.^19^ Thus, data might be incomplete for these individuals, and the findings might not be generalisable to individuals such as these. Furthermore, there were variations in follow-up length and age at age of follow-up between individuals in the study. To account for these issues, we adjusted for birth year and allowed disorder prevalences to vary in full-siblings and half-siblings. However, if the changes in diagnostic practices and register coverage changed the phenotypes and their aetiologies, or have had differential effects on conditions, biases might have remained.

Adult ADHD only became a commonly diagnosed disorder in Sweden in the past decade, which probably explains the lower prevalence in our study (1·3%) than in previous survey studies in adults.^3^, ^4^ The resulting misclassification of ADHD cases might dilute the estimated associations, especially in middle and late adulthood, potentially underestimating associations with late-onset conditions the most. Furthermore, ADHD is associated with premature mortality.^40^ Individuals who do not reach old age are less likely to develop late-onset conditions, which might further lead to underestimated associations between ADHD and late-onset conditions. Replication using other data sources would strengthen the inferences from our study.

The heritability of a few health conditions (eg, epilepsy and type 1 diabetes) was slightly lower than in previous reports.^18^, ^41^ These lower heritability rates might be the result of bias from incomplete coverage of diseases and varying follow-up lengths. Another possible reason could be the older age-range, as previous research has reported negative associations between disease onset age and heritability.^41^, ^42^, ^43^ To ensure generalisability of the findings from this Swedish sibling cohort, further replication is needed in other populations from other nationalities. For the quantitative genetic models, we assumed that full-siblings and maternal half-siblings share their common environment to the same degree. We acknowledge that this assumption is a simplification, but previous analyses using Swedish tax records showed that nearly all full-siblings and maternal half-siblings were registered to the same household during childhood.^30^

In conclusion, we showed that adults with ADHD are at increased risk of a range of physical conditions, across circulatory, metabolic, gastrointestinal, genitourinary, musculoskeletal, nervous system, respiratory, and skin diseases. Most physical conditions showed familial associations with ADHD (mainly from genetic factors). Our findings highlight the need for rigorous medical assessment and care in adult patients with ADHD, and suggest long-term consequences with age-related diseases. We further shed light on the aetiological underpinnings of physical conditions in ADHD, which could guide future research that aims to identify shared biological mechanisms, or modifiable risk factors that contribute to risk of physical conditions in patients with ADHD.

## Data sharing

The Public Access to Information and Secrecy Act in Sweden prohibits us from making individual-level data publicly available. Researchers who are interested in replicating our work can apply for individual-level data through Statistics Sweden at: https://www.scb.se/en/services/guidance-for-researchers-and-universities/.

## Declaration of interests

EDR reports grants from FORTE, Ingrid and Thurings Stiftelse, during the conduct of the study; and personal speaker fees from Shire Sweden, a Takeda Pharmaceuticals company, outside of the submitted work. HL has served as a speaker for Evolan Pharma and Shire Sweden and has received research grants from Shire Sweden, outside of the submitted work. IB reports grants from The Swedish Brain Foundation, during the conduct of the study. ML reports personal fees from Johnson and Johnson, and grants from the EU's Horizon 2020, outside of the submitted work. SC reports honoraria and reimbursement for travel and accommodation expenses for lectures from the following non-profit associations: Association for Child and Adolescent Central Health, Canadian ADHD Alliance Resource, British Association of Pharmacology, and Healthcare Convention for Educational Activity on ADHD, during the conduct of the study. SVF has received income, potential income, travel expenses, or continuing education support or research support from Takeda, OnDosis, Tris, Otsuka, Arbor, Alcobra, Aveksham, Enzymotec, Neurovance, Vallon, KemPharm-Corium, Lundbeck, Ironshore, Rhodes, Akili Interactive Labs, Sunovion, Supernus, and Genomind; has US patent US20130217707 A1 for the use of sodium-hydrogen exchange inhibitors in the treatment of ADHD; receives royalties from books published by Guilford Press, Oxford University Press, and Elsevier; and is programme director of www.adhdinadults.com, outside of the submitted work. All other authors declare no competing interests. EDR affirms that this manuscript is an honest, accurate, and transparent account of the study being reported; that no important aspects of the study have been omitted; and that any discrepancies from the study as planned (and, if relevant, registered) have been explained.
