# Supplementary material for: Mapping phenotypic and aetiological associations between ADHD and physical conditions in adulthood in Sweden: a genetically informed register study
Source: Lancet Psychiatry. 2021 Sep;8(9):774–83. doi: 10.1016/S2215-0366(21)00171-1 (PMC8376653; doi:10.1016/S2215-0366(21)00171-1)
Supplement: Supplementary appendix [file mmc1.pdf]

THE LANCET
Psychiatry

Supplementary appendix

This appendix formed part of the original submission and has been peer reviewed. We post it as supplied by the authors.

Supplement to: Du Rietz E, Brikell I, Butwicka A, et al. Mapping phenotypic and aetiological associations between ADHD and physical conditions in adulthood in Sweden: a genetically informed register study. *Lancet Psychiatry* 2021; published online July 6. http://dx.doi.org/10.1016/S2215-0366(21)00171-1.

Appendix

Du Rietz E, Brikell I, Butwicka A, Leone M, Chang Z, Cortese S, D'Onofrio BM, Hartman C, Lichtenstein P, Faraone SV, Kuja-Halkola, R, Larsson H. Mapping phenotypic and aetiological associations between ADHD and physical conditions in adulthood in Sweden: a genetically informed register study. *Lancet Psychiatry* 2021.

Table of Contents

eTable 1. ICD codes used for physical conditions 2

eTable 2. Prevalence of physical conditions in individuals with and without ADHD 4

eTable 3. Prevalence of physical conditions in full- and maternal-half siblings of individuals with and without ADHD: exposure persons born 1932-1944 5

eTable 4. Prevalence of physical conditions in full- and maternal-half siblings of individuals with and without ADHD: exposure persons born 1945-1957 6

eTable 5. Prevalence of physical conditions in full- and maternal-half siblings of individuals with and without ADHD: exposure persons born 1958-1970 7

eTable 6. Prevalence of physical conditions in full- and maternal-half siblings of individuals with and without ADHD: exposure persons born 1971-1983 8

eTable 7. Prevalence of physical conditions in full- and maternal-half siblings of individuals with and without ADHD: exposure persons born 1984-1995 9

eTable 8. Associations between ADHD and physical disease groups within-individuals and between full- and maternal half-siblings 10

eTable 9. Associations between ADHD and physical conditions across full-siblings, adjusting for ADHD status in co-sibling (outcome person) 11

Table 10. Associations between ADHD and physical conditions within-individuals and between full- and maternal half-siblings: Including only sibling pairs born within 10 years apart 12

eTable 11. Prevalence of conditions and associations between ADHD and physical conditions within-individuals and between full- and maternal half-siblings: in males (N = 2,449,146) 13

eTable 12. Prevalence of conditions and associations between ADHD and physical conditions within-individuals and between full- and maternal half-siblings: in females (N = 2,340,653) 14

eTable 13. Univariate ACE models with bootstrap 95% confidence intervals 15

eTable 14. A, C and E correlations with bootstrap 95% confidence intervals 16

eTable 15. Explained variance from A, C and E with bootstrap 95% confidence intervals 17

eTable 1. ICD codes used for physical conditions

	ICD 10 code	ICD 9 code	ICD 8 code
**CIRCULATORY SYSTEM**			
Hypertension	I10-I13, I15	401- 405	400-404
Ischemic heart disease	I20-25	410-414	410-414
Pulmonary heart disease	I26-I28	415, 416, 417	426, 450
Atrial fibrillation and other cardiac arrhythmia	I48-149	426A, 427D-E, 427G, 427W-X	426·01-426·09, 427·90-427·99
Heart failure	I50	428	42700, 42719, 42799
Cerebrovascular disease	I60-I69, G45, G46	344W, 352G, 430,431,432, 433, 434,436, 437	356·00-356·09, 430,431,432, 433,434,436,437·99, 438·99, 440·20
Peripheral vascular disease	I70-74, I77	421A, 440, 441, 442, 443, 444, 447	421·00, 440, 441, 442, 443, 444, 447·00-09
**ENDOCRINE & METABOLIC DISEASE**			
Type 1 diabetes mellitus	E10	250	250
Type 2 diabetes mellitus	E11	-	-
Thyroid disorder	E00-E05, E06, E07	240, 241, 242, 243, 244, 245, 246A, 246B, 246C, 246W	240, 241, 2420, 2421, 2422, 243, 244, 245, 246
Obesity	E65-E66	278A, 278B	27799
Gout	M10	274	274
**GASTROINTESTINAL SYSTEM**			
Celiac disease	K90·0	579A	269·00
Ulcer/chronic gastritis	K25-28, K29·3-29·5	531, 532, 533, 534, 535B	531, 532, 533, 534, 53503
Acute appendicitis	K35	540	540
Non-alcoholic fatty liver disease	K75·8, K76·0	573W, 571W	5719, 57308
Alcohol-related liver diseases	K70	571A–D	571·00, 571·01
IBD	K50-51	555, 556	5630, 5631, 569
Gallstone disease (including acute pancreatitis)	K80, K85·1	574, 577A	574, 5770
**GENITOURINARY SYSTEM**			
Glomerular disease	N00-N08	580-583	580-584
Kidney infections	N10-12	590	590
Urolithiasis (calculus on kidney, ureter and lower urinary tract)	N20-21	592A-X, 593X, 594A-X	592·00-594·08
**MUSCULOSKELETAL SYSTEM**			
Rheumatoid arthritis	M05, M06·0, M06·2, M06·3, M06·8, M06·9, M12·3	714A-C, 714W, 719D	71210, 71220, 71238, 71339
Arthrosis (osteoarthritis)	M15-19	715	713
Systemic connective tissue disorders	M30-M36	446, 447G, 447W, 710, 725, 711B, 729D/E/X, 728F, 713A/C/G/H, 716X	446, 447, 734, 7179, 729, 7339, 732, 711, 7149, 715, 718
Dorsalgia (back pain)	M54	723B/E/G, 724B/C/D/E/F/X	726, 7270, 7280, 728, 352, 353
**DISEASE OF THE NERVOUS SYSTEM**			
Parkinson disease and parkinsonism	G20, G21·2, G21·3, G21·8, G21·9, G23·1, G23·2, G23·8, G23·9, G25·9	3320, 3321, 3330	34200, 34208, 34209
Dementia	G30, G31·1, G31·8A, F00-F03, F05·1	290A, 290B, 290E, 290W, 290X, 294B, 331A, 331B, 331C, 331X	29000, 29010, 29011, 29019, 29300, 2931
Epilepsy	G40-41	345	345
Migraine	G43	346A/B/X/W	34609
Sleep disorder*	G47·0/1/2/3/8/9	780F	7806, 3064
**DISEASES OF RESPIRATORY SYSTEM**			
Asthma	J45, J46	493	493
Chronic Obstructive Pulmonary Disease	J40-44	490–492, 496	490, 491, 492

**DISEASES OF THE SKIN**			
Atopic dermatitis (eczema)	L20	691W	691
Psoriasis	L40	696	696

*Narcolepsy excluded in sleep disorders

eTable 2. Prevalence of physical conditions in individuals with and without ADHD

	Individuals with ADHD (n=61,960; 1.29%)		Individuals without ADHD (n=4,727,839)	
**Median birth year**	**1982**		**1964**	
	Crude prevalence	Age-standardised prevalence	Crude prevalence	Age-standardised prevalence
**CIRCULATORY SYSTEM**				
Hypertension	2,339 (3·78%)	12.99%	459,726 (9·72%)	9.63%
Ischemic heart disease	603 (0·97%)	4.93%	184,134 (3·89%)	3.85%
Pulmonary disease	583 (0·94%)	1.27%	37,805 (0·80%)	0.80%
Atrial fibrillation	903 (1·46%)	4.24%	168,083 (3·56%)	3.52%
Heart failure	329 (0·53%)	2.40%	70,742 (1·49%)	1.48%
Stroke	557 (0·90%)	3.37%	96,833 (2·05%)	2.03%
Peripheral vascular disease	356 (0·57%)	1.54%	61,830 (1·31%)	1.29%
**ENDOCRINE/METABOLIC**				
Type 1 diabetes mellitus	923 (1·49%)	2.41%	79,818 (1·69%)	1.68%
Type 2 diabetes mellitus	1,123 (1·81%)	4.79%	153,086 (3·23%)	3.21%
Thyroid disorder	1,783 (2·88%)	5.25%	148,078 (3·13%)	3.11%
Obesity	3,068 (4·95%)	5.65%	110,700 (2·34%)	2.34%
Gout	151 (0·24%)	0.74%	20,535 (0·43%)	0.43%
**GASTROINTESTINAL**				
Celiac disease	345 (0·56%)	0.56%	18,607 (0·39%)	0.39%
Ulcer/chronic gastritis	829 (1·34%)	3.18%	72,387 (1·53%)	1.52%
Acute appendicitis	1,865 (3·01%)	4.16%	152,707 (3·23%)	3.22%
Fatty liver disease	117 (0·19%)	0.35%	5,708 (0·12%)	0.12%
Alcohol-related liver disease	184 (0·30%)	0.64%	10,164 (0·21%)	0.21%
IBD	799 (1·29%)	1.90%	63,991 (1·35%)	1.35%
Gallstone disease	2,438 (3·93%)	6.80%	211,093 (4·46%)	4.44%
**GENITOURINARY**				
Glomerular disease	343 (0·56%)	0.88%	22,623 (0·48%)	0.48%
Urolithiasis	1,395 (2·25%)	3.84%	127,187 (2·69%)	2.67%
Kidney infections	1,177 (1·90%)	2.87%	60,118 (1·27%)	1.27%
**MUSCULOSKELETAL**				
Rheumatoid arthritis	242 (0·39%)	0.84%	42,743 (0·90%)	0.90%
Arthrosis	1,741 (2·81%)	8.77%	320,676 (6·78%)	6.72%
Connective tissue disease	627 (1·01%)	2.23%	68,238 (1·44%)	1.43%
Dorsalgia (back pain)	5,825 (9·40%)	13.51%	292,251 (6·18%)	6.15%
**NERVOUS SYSTEM**				
Parkinson’s disease	47 (0·08%)	0.33%	12,522 (0·26%)	0.26%
Dementia	73 (0·12%)	0.78%	20,656 (0·44%)	0.43%
Epilepsy	1,720 (2·78%)	3.34%	60,422 (1·28%)	1.27%
Migraine	1,519 (2·45%)	2.84%	68,907 (1·46%)	1.46%
Sleep disorder	3,652 (5·89%)	9.73%	119,328 (2·52%)	2.51%
**RESPIRATORY**				
Asthma	3,496 (5·64%)	6.68%	140,332 (2·97%)	2.96%
COPD	695 (1·12%)	3.62%	74,173 (1·57%)	1.55%
**SKIN**				
Eczema	1,002 (1·62%)	1.55%	49,055 (1·04%)	1.04%
Psoriasis	1,061 (1·71%)	2.72%	88,578 (1·87%)	1.86%

IBD: Irritable bowel syndrome, COPD: chronic obstructive pulmonary disease.

eTable 3. Prevalence of physical conditions in full- and maternal-half siblings of individuals with and without ADHD: exposure persons born 1932-1944

	**Full-siblings of individuals:**		**Maternal half-siblings of individuals:**	
	with ADHD (n=799; 0·07%)	without ADHD (n=1,187,479)	with ADHD (n=17; 0·04%)	without ADHD (n=38,809)
**ADHD**	7 (0·88%)	1,265 (0·11%)	0	152 (0·39%)
**CIRCULATORY SYSTEM**				
Hypertension	212 (26·53%)	350,590 (29·52%)	3 (17·65%)	9,487 (24·45%)
Ischemic heart disease	114 (14·27%)	172,334 (14·51%)	1 (5·88%)	4,548 (11·72%)
Pulmonary disease	10 (1·25%)	26,631 (2·24%)	0	714 (1·84%)
Atrial fibrillation	88 (11·01%)	131,087 (11·04%)	4 (23·53%)	3,430 (8·84%)
Heart failure	49 (6·13%)	67,292 (5·67%)	1 (5·88%)	1,867 (4·81%)
Stroke	46 (5·76%)	83,592 (7·04%)	1 (5·88%)	2,143 (5·52%)
Peripheral vascular disease	38 (4·76%)	54,323 (4·57%)	1 (5·88%)	1,317 (3·39%)
**ENDOCRINE/METABOLIC**				
Type 1 diabetes mellitus	29 (3·63%)	41,816 (3·52%)	1 (5·88%)	1,284 (3·31%)
Type 2 diabetes mellitus	72 (9·01%)	115,321 (9·71%)	1 (5·88%)	3,531 (9·10%)
Thyroid disorder	49 (6·13%)	73,201 (6·16%)	2 (11·76%)	2,196 (5·66%)
Obesity	34 (4·26%)	29,525 (2·49%)	1 (5·88%)	1,274 (3·28%)
Gout	12 (1·50%)	14,527 (1·22%)	0	413 (1·06%)
**GASTROINTESTINAL**				
Celiac disease	2 (0·25%)	5,459 (0·46%)	0	138 (0·36%)
Ulcer/chronic gastritis	30 (3·75%)	54,788 (4·61%)	1 (5·88%)	1,620 (4·17%)
Acute appendicitis	29 (3·63%)	40,024 (3·37%)	0	1,452 (3·74%)
Fatty liver disease	3 (0·38%)	2,658 (0·22%)	0	90 (0·23%)
Alcohol-related liver disease	4 (0·50%)	5,809 (0·49%)	1 (5·88%)	241 (0·62%)
IBD	12 (1·50%)	20,093 (1·69%)	0	666 (1·72%)
Gallstone disease	69 (8·64%)	105,626 (8·89%)	2 (11·76%)	3,278 (8·45%)
**GENITOURINARY**				
Glomerular disease	4 (0·50%)	10,016 (0·84%)	0	363 (0·94%)
Urolithiasis	42 (5·26%)	61,341 (5·17%)	1 (5·88%)	1,929 (4·97%)
Kidney infections	24 (3·00%)	26,925 (2·27%)	1 (5·88%)	824 (2·12%)
**MUSCULOSKELETAL**				
Rheumatoid arthritis	19 (2·38%)	27,030 (2·28%)	0	754 (1·94)
Arthrosis	144 (18·02%)	210,130 (17·70%)	1 (5·88%)	5,889 (15·17%)
Connective tissue disease	26 (3·25%)	42,658 (3·59%)	1 (5·88%)	1,191 (3·07%)
Dorsalgia (back pain)	84 (10·51%)	112,071 (9·44%)	2 (11·76%)	3,865 (9·96%)
**NERVOUS SYSTEM**				
Parkinson’s disease	6 (0·75%)	12,180 (1·03%)	0	307 (0·79%)
Dementia	14 (1·75%)	25,165 (2·12%)	0	569 (1·47%)
Epilepsy	20 (2·50%)	23,379 (1·97%)	0	825 (2·13%)
Migraine	17 (2·13%)	15,262 (1·29%)	0	511 (1·32%)
Sleep disorder	35 (4·38%)	52,305 (4·40%)	0	1,668 (4·30%)
**RESPIRATORY**				
Asthma	41 (5·13%)	47,520 (4·00%)	2 (11·76%)	1,527 (3·93%)
COPD	58 (7·26%)	63,922 (5·38%)	1 (5·88%)	1,910 (4·92%)
**SKIN**				
Eczema	6 (0·75%)	6,439 (0·54%)	0	248 (0·64%)
Psoriasis	29 (3·63%)	35,755 (3·01%)	0	1,229 (3·17%)

IBD: Irritable bowel syndrome, COPD: chronic obstructive pulmonary disease. N: Not unique individuals as individuals are repeated if they have multiple siblings.

eTable 4. Prevalence of physical conditions in full- and maternal-half siblings of individuals with and without ADHD: exposure persons born 1945-1957

	Full-siblings of individuals:		Maternal half-siblings of individuals:	
	with ADHD (n=4,938; 0·25%)	without ADHD (n=1,961,380)	with ADHD (n=781; 0·53%)	without ADHD (n=146,514)
**ADHD**	115 (2·33%)	5,840 (0·30%)	17 (2·18%)	1,347 (0·92%)
**CIRCULATORY SYSTEM**				
Hypertension	776 (15·71%)	376,483 (19·19%)	109 (13·96%)	23,474 (16·02%)
Ischemic heart disease	315 (6·38%)	160,056 (8·16%)	45 (5·76%)	9,624 (6·57%)
Pulmonary disease	50 (1·01%)	27,395 (1·40%)	6 (0·77%)	1,936 (1·32%)
Atrial fibrillation	263 (5·33%)	123,176 (6·28%)	39 (4·99%)	7,615 (5·20%)
Heart failure	123 (2·49%)	55,437 (2·83%)	16 (2·05%)	3,686 (2·52%)
Stroke	166 (3·36%)	76,876 (3·92%)	22 (2·82%)	4,949 (3·38%)
Peripheral vascular disease	104 (2·11%)	49,096 (2·50%)	16 (2·05%)	3,253 (2·22%)
**ENDOCRINE/METABOLIC**				
Type 1 diabetes mellitus	107 (2·17%)	50,194 (2·56%)	22 (2·82%)	3,816 (2·60%)
Type 2 diabetes mellitus	244 (4·94%)	123,761 (6·31%)	37 (4·74%)	9,009 (6·15%)
Thyroid disorder	238 (4·82%)	91,002 (4·64%)	30 (3·84%)	6,578 (4·49%)
Obesity	134 (2·71%)	51,705 (2·64%)	35 (4·48%)	5,788 (3·95%)
Gout	42 (0·85%)	15,482 (0·79%)	7 (0·90%)	1,155 (0·79%)
**GASTROINTESTINAL**				
Celiac disease	15 (0·30%)	7,384 (4·02%)	2 (0·26%)	517 (0·35%)
Ulcer/chronic gastritis	163 (3·30%)	57,365 (2·92%)	17 (2·18%)	4,472 (3·05%)
Acute appendicitis	217 (4·39%)	78,801 (4·02%)	37 (4·74%)	5,978 (4·08%)
Fatty liver disease	7 (0·14%)	3,985 (0·20%)	2 (0·26%)	349 (0·24%)
Alcohol-related liver disease	31 (0·63%)	8,112 (0·41%)	5 (0·64%)	857 (0·58%)
IBD	91 (1·84%)	32,708 (1·67%)	15 (1·92%)	2,588 (1·77%)
Gallstone disease	370 (7·49%)	138,909 (7·08%)	58 (7·43%)	11·152 (7·61%)
**GENITOURINARY**				
Glomerular disease	39 (0·79%)	14,848 (0·76%)	10 (1·28%)	1,177 (0·80%)
Urolithiasis	219 (4·43%)	84,825 (4·32%)	25 (3·20%)	5,954 (4·06%)
Kidney infections	110 (2·23%)	33,697 (1·72%)	22 (2·82%)	2,810 (1·92%)
**MUSCULOSKELETAL**				
Rheumatoid arthritis	72 (1·46%)	31,520 (1·61%)	10 (1·28%)	2,274 (1·55%)
Arthrosis	616 (12·47%)	255,176 (13·01%)	69 (8·83%)	16,034 (10·94%)
Connective tissue disease	115 (2·33%)	48,963 (2·50%)	19 (2·43%)	3,463 (2·36%)
Dorsalgia (back pain)	528 (10·69%)	167,921 (8·56%)	85 (10·88%)	14,866 (10·15%)
**NERVOUS SYSTEM**				
Parkinson's disease	25 (0·51%)	10,007 (0·51%)	4 (0·51%)	469 (0·32%)
Dementia	27 (0·55%)	14,930 (0·76%)	5 (0·64%)	759 (0·52%)
Epilepsy	88 (1·78%)	33,180 (1·69%)	14 (1·79%)	2,788 (1·90%)
Migraine	88 (1·78%)	28,772 (1·47%)	12 (1·54%)	2,596 (1·77%)
Sleep disorder	231 (4·68%)	84,842 (4·33%)	46 (5·89%)	5,940 (4·05%)
**RESPIRATORY**				
Asthma	213 (4·31%)	66,891 (3·41%)	36 (4·61%)	5,965 (4·07%)
COPD	178 (3·60%)	61,160 (3·12%)	33 (4·23%)	5,125 (3·50%)
**SKIN**				
Eczema	38 (0·77%)	13,710 (0·70%)	3 (0·38%)	1,180 (0·81%)
Psoriasis	152 (3·08%)	54,200 (2·76%)	25 (3·20%)	4,321 (2·95%)

IBD: Irritable bowel syndrome, COPD: chronic obstructive pulmonary disease. N: Not unique individuals as individuals are repeated if they have multiple siblings.

eTable 5. Prevalence of physical conditions in full- and maternal-half siblings of individuals with and without ADHD: exposure persons born 1958-1970

	Full-siblings of individuals:		Maternal half-siblings of individuals:	
	with ADHD (n=13,900; 0·83%)	without ADHD (n=1,654,069)	with ADHD (n=5,117; 2·07%)	without ADHD (n=241,591)
**ADHD**	613 (4·41%)	12,807 (0·77%)	270 (5·28%)	4,978 (2·06%)
**CIRCULATORY SYSTEM**				
Hypertension	1,074 (7·73%)	121,261 (7·33%)	351 (6·86%)	18,893 (7·82%)
Ischemic heart disease	387 (2·78%)	37,797 (2·29%)	115 (2·25%)	6,504 (2·69%)
Pulmonary disease	104 (0·75%)	10,092 (0·61%)	41 (0·80%)	1,791 (0·74%)
Atrial fibrillation	325 (2·34%)	39,346 (2·38%)	111 (2·17%)	6,010 (2·49%)
Heart failure	112 (0·81%)	12,552 (0·76%)	55 (1·07%)	2,349 (0·97%)
Stroke	217 (1·56%)	20,819 (1·26%)	75 (1·47%)	3,782 (1·57%)
Peripheral vascular disease	115 (0·83%)	12,595 (0·76%)	52 (1·02%)	2,439 (1·01%)
**ENDOCRINE/METABOLIC**				
Type 1 diabetes mellitus	245 (1·76%)	24,457 (1·48%)	66 (1·29%)	4,089 (1·69%)
Type 2 diabetes mellitus	417 (3·00%)	40,892 (2·47%)	138 (2·70%)	7,370 (3·05%)
Thyroid disorder	497 (3·58%)	51,567 (3·12%)	184 (3·60%)	8,057 (3·33%)
Obesity	566 (4·07%)	45,280 (2·74%)	243 (4·75%)	10,412 (4·31%)
Gout	59 (0·42%)	5,654 (0·34%)	20 (0·39%)	1,022 (0·42%)
**GASTROINTESTINAL**				
Celiac disease	52 (0·37%)	5,642 (0·34%)	21 (0·41%)	828 (0·34%)
Ulcer/chronic gastritis	223 (1·60%)	20,188 (1·22%)	102 (1·99%)	3,865 (1·60%)
Acute appendicitis	616 (4·43%)	70,536 (4·26%)	193 (3·77%)	9,184 (3·80%)
Fatty liver disease	31 (0·22%)	2,165 (0·13%)	10 (0·20%)	419 (0·17%)
Alcohol-related liver disease	55 (0·40%)	3,157 (0·19%)	22 (0·43%)	872 (0·36%)
IBD	228 (1·64%)	24,764 (1·50%)	87 (1·70%)	3,738 (1·55%)
Gallstone disease	741 (5·33%)	79,712 (4·82%)	306 (5·98%)	13,735 (5·69%)
**GENITOURINARY**				
Glomerular disease	96 (0·69%)	8,461 (0·51%)	12 (0·23%)	1,315 (0·54%)
Urolithiasis	411 (2·96%)	47,700 (2·88%)	154 (3·01%)	7,276 (3·01%)
Kidney infections	213 (1·53%)	19,983 (1·21%)	82 (1·60%)	3,953 (1·64%)
**MUSCULOSKELETAL**				
Rheumatoid arthritis	121 (0·87%)	13,834 (0·84%)	48 (0·94%)	2,191 (0·91%)
Arthrosis	961 (6·91%)	104,168 (6·30%)	293 (5·73%)	14,439 (5·98%)
Connective tissue disease	190 (1·37%)	21,444 (1·30%)	68 (1·33%)	3,414 (1·41%)
Dorsalgia (back pain)	1,418 (10·20%)	119,983 (7·25%)	506 (9·89%)	21,180 (8·77%)
**NERVOUS SYSTEM**				
Parkinson's disease	16 (0·12%)	1,973 (0·12%)	8 (0·16%)	267 (0·11%)
Dementia	14 (0·10%)	1,795 (0·11%)	5 (0·10%)	326 (0·13%)
Epilepsy	253 (1·82%)	21,292 (1·29%)	96 (1·88%)	3,956 (1·64%)
Migraine	302 (2·17%)	28,604 (1·73%)	121 (2·36%)	4,798 (1·99%)
Sleep disorder	583 (4·19%)	48,667 (2·94%)	160 (3·13%)	7,470 (3·09%)
**RESPIRATORY**				
Asthma	526 (3·78%)	47,360 (2·86%)	231 (4·51%)	8,982 (3·72%)
COPD	246 (1·77%)	15,491 (0·94%)	92 (1·80%)	4,001 (1·66%)
**SKIN**				
Eczema	147 (1·06%)	15,668 (0·95%)	65 (1·27%)	2,518 (1·04%)
Psoriasis	303 (2·18%)	34,108 (2·06%)	142 (2·78%)	5,957 (2·47%)

IBD: Irritable bowel syndrome, COPD: chronic obstructive pulmonary disease. N: Not unique individuals as individuals are repeated if they have multiple siblings.

eTable 6. Prevalence of physical conditions in full- and maternal-half siblings of individuals with and without ADHD: exposure persons born 1971-1983

	Full-siblings of individuals:		Maternal half-siblings of individuals:	
	with ADHD (n=19,235; 1·37%)	without ADHD (n=1,385,418)	with ADHD (n=10,672; 3·72%)	without ADHD (n=276,382)
**ADHD**	1,456 (7·57%)	18,199 (1·31%)	937 (8·78%)	9,855 (3·57%)
**CIRCULATORY SYSTEM**				
Hypertension	363 (1·89%)	20,202 (1·46%)	242 (2·27%)	6,956 (2·52%)
Ischemic heart disease	71 (0·37%)	3,405 (0·25%)	71 (0·67%)	1,669 (0·60%)
Pulmonary disease	56 (0·29%)	3,435 (0·25%)	42 (0·39%)	1,057 (0·38%)
Atrial fibrillation	185 (0·96%)	12,841 (0·93%)	103 (0·97%)	3,091 (1·12%)
Heart failure	45 (0·23%)	2,038 (0·15%)	28 (0·26%)	763 (0·28%)
Stroke	79 (0·41%)	4,215 (0·30%)	56 (0·52%)	1,464 (0·53%)
Peripheral vascular disease	55 (0·29%)	3,222 (0·23%)	31 (0·29%)	921 (0·33%)
**ENDOCRINE/METABOLIC**				
Type 1 diabetes mellitus	184 (0·96%)	12,257 (0·88%)	109 (1·02%)	3,099 (1·12%)
Type 2 diabetes mellitus	174 (0·90%)	8,300 (0·60%)	109 (1·02%)	3,106 (1·12%)
Thyroid disorder	491 (2·55%)	29,378 (2·12%)	241 (2·26%)	5,825 (2·11%)
Obesity	694 (3·61%)	31,868 (2·30%)	470 (4·40%)	9,771 (3·54%)
Gout	25 (0·13%)	1,479 (0·11%)	16 (0·15%)	469 (0·17%)
**GASTROINTESTINAL**				
Celiac disease	87 (0·45%)	5,545 (0·40%)	41 (0·38%)	1,052 (0·38%)
Ulcer/chronic gastritis	116 (0·60%)	6,712 (0·48%)	96 (0·90%)	1,936 (0·70%)
Acute appendicitis	609 (3·17%)	42,174 (3·04%)	307 (2·88%)	8,150 (2·95%)
Fatty liver disease	17 (0·09%)	882 (0·06%)	7 (0·07%)	239 (0·09%)
Alcohol-related liver disease	12 (0·06%)	442 (0·03%)	13 (0·12%)	262 (0·09%)
IBD	222 (1·15%)	16,825 (1·21%)	122 (1·14%)	3,218 (1·16%)
Gallstone disease	623 (3·24%)	35,984 (2·60%)	397 (3·72%)	9,588 (3·47%)
**GENITOURINARY**				
Glomerular disease	80 (0·42%)	3,921 (0·28%)	42 (0·39%)	953 (0·34%)
Urolithiasis	358 (1·86%)	21,782 (1·57%)	201 (1·88%)	4,979 (1·80%)
Kidney infections	248 (1·29%)	12,523 (0·90%)	164 (1·54%)	3,468 (1·25%)
**MUSCULOSKELETAL**				
Rheumatoid arthritis	66 (0·34%)	4,923 (0·36%)	34 (0·32%)	1,129 (0·41%)
Arthrosis	336 (1·75%)	21,983 (1·59%)	201 (1·88%)	6,591 (2·38%)
Connective tissue disease	123 (0·64%)	7,341 (0·53%)	76 (0·71%)	1,927 (0·70%)
Dorsalgia (back pain)	1,282 (6·66%)	67,543 (4·88%)	747 (7·00%)	17,416 (6·30%)
**NERVOUS SYSTEM**				
Parkinson's disease	1 (0·005%)	296 (0·02%)	5 (0·05%)	91 (0·03%)
Dementia	5 (0·03%)	167 (0·01%)	2 (0·02%)	83 (0·03%)
Epilepsy	248 (1·29%)	13,949 (1·01%)	171 (1·60%)	3,612 (1·31%)
Migraine	437 (2·27%)	22,578 (1·63%)	218 (2·04%)	4,990 (1·81%)
Sleep disorder	414 (2·15%)	17,742 (1·28%)	245 (2·30%)	5,046 (1·83%)
**RESPIRATORY**				
Asthma	709 (3·69%)	36,266 (2·62%)	427 (4·01%)	9,247 (3·35%)
COPD	60 (0·31%)	2,819 (0·20%)	56 (0·52%)	1,308 (0·47%)
**SKIN**				
Eczema	259 (1·35%)	19,108 (1·38%)	129 (1·21%)	3,408 (1·23%)
Psoriasis	318 (1·65%)	17,803 (1·29%)	166 (1·56%)	4,172 (1·51%)

IBD: Irritable bowel syndrome, COPD: chronic obstructive pulmonary disease. N: Not unique individuals as individuals are repeated if they have multiple siblings.

eTable 7. Prevalence of physical conditions in full- and maternal-half siblings of individuals with and without ADHD: exposure persons born 1984-1995

	Full-siblings of individuals:		Maternal half-siblings of individuals:	
	with ADHD (n=33,416; 2·37%)	without ADHD (n=1,377,780)	with ADHD (n=12,529; 5·73%)	without ADHD (n=206,076)
**ADHD**	3,963 (11·86%)	28,023 (2·03%)	1,422 (11·35%)	10,138 (4·92%)
**CIRCULATORY SYSTEM**				
Hypertension	146 (0·44%)	4,557 (0·33%)	103 (0·82%)	1,973 (0·96%)
Ischemic heart disease	22 (0·07%)	465 (0·03%)	14 (0·11%)	287 (0·14%)
Pulmonary disease	30 (0·09%)	1,407 (0·10%)	23 (0·18%)	457 (0·22%)
Atrial fibrillation	144 (0·43%)	5,227 (0·38%)	103 (0·82%)	1,510 (0·73%)
Heart failure	11 (0·03%)	679 (0·05%)	13 (0·10%)	239 (0·12%)
Stroke	45 (0·13%)	1,421 (0·10%)	24 (0·19%)	544 (0·26%)
Peripheral vascular disease	42 (0·13%)	1,398 (0·10%)	25 (0·20%)	402 (0·20%)
**ENDOCRINE/METABOLIC**				
Type 1 diabetes mellitus	297 (0·89%)	9,984 (0·72%)	96 (0·77%)	1,790 (0·87%)
Type 2 diabetes mellitus	90 (0·27%)	2,535 (0·18%)	61 (0·49%)	1,021 (0·50%)
Thyroid disorder	422 (1·26%)	14,655 (1·06%)	224 (1·79%)	3,525 (1·71%)
Obesity	813 (2·43%)	18,630 (1·35%)	485 (3·87%)	6,318 (3·07%)
Gout	16 (0·05%)	467 (0·03%)	9 (0·07%)	166 (0·08%)
**GASTROINTESTINAL**				
Celiac disease	173 (0·52%)	6,440 (0·47%)	47 (0·38%)	869 (0·42%)
Ulcer/chronic gastritis	104 (0·31%)	3,217 (0·23%)	58 (0·46%)	885 (0·43%)
Acute appendicitis	515 (1·54%)	20,335 (1·48%)	306 (2·44%)	4,967 (2·41%)
Fatty liver disease	9 (0·03%)	372 (0·03%)	5 (0·04%)	105 (0·05%)
Alcohol-related liver disease	2 (0·01%)	79 (0·01%)	6 (0·05%)	71 (0·03%)
IBD	283 (0·85%)	11,186 (0·81%)	124 (0·99%)	2,096 (1·02%)
Gallstone disease	442 (1·32%)	13,277 (0·96%)	329 (2·63%)	5,072 (2·46%)
**GENITOURINARY**				
Glomerular disease	57 (0·17%)	2,172 (0·16%)	43 (0·34%)	545 (0·26%)
Urolithiasis	309 (0·92%)	11,054 (0·80%)	177 (1·41%)	2,874 (1·39%)
Kidney infections	320 (0·96%)	9,647 (0·70%)	139 (1·11%)	2,310 (1·12%)
**MUSCULOSKELETAL**				
Rheumatoid arthritis	41 (0·12%)	1,794 (0·13%)	44 (0·35%)	581 (0·28%)
Arthrosis	126 (0·38%)	5,583 (0·41%)	98 (0·78%)	2,008 (0·97%)
Connective tissue disease	140 (0·42%)	4,015 (0·29%)	60 (0·48%)	963 (0·47%)
Dorsalgia (back pain)	1,146 (3·43%)	35,735 (2·59%)	737 (5·88%)	10,077 (4·89%)
**NERVOUS SYSTEM**				
Parkinson's disease	3 (0·01%)	83 (0·01%)	2 (0·02%)	29 (0·01%)
Dementia	2 (0·01%)	70 (0·01%)	5 (0·04%)	14 (0·01%)
Epilepsy	382 (1·14%)	10,468 (0·76%)	166 (1·32%)	2,373 (1·15%)
Migraine	462 (1·38%)	13,234 (0·96%)	221 (1·76%)	3,470 (1·68%)
Sleep disorder	385 (1·15%)	8,020 (0·58%)	216 (1·72%)	2,776 (1·34%)
**RESPIRATORY**				
Asthma	1,052 (3·15%)	30,104 (2·18%)	460 (3·67%)	6,539 (3·17%)
COPD	45 (0·13%)	1,065 (0·08%)	27 (0·22%)	404 (0·20%)
**SKIN**				
Eczema	442 (1·32%)	17,371 (1·26%)	190 (1·52%)	2,935 (1·42%)
Psoriasis	299 (0·89%)	9,370 (0·68%)	167 (1·33%)	2,427 (1·18%)

IBD: Irritable bowel syndrome, COPD: chronic obstructive pulmonary disease. N: Not unique individuals as individuals are repeated if they have multiple siblings.

eTable 8. Associations between ADHD and physical disease groups within-individuals and between full- and maternal half-siblings

Disease group	OR (95% CIs)			P value
	Within ind.	Full sibs	Half sibs	Full vs half sibs
NERVOUS SYSTEM	3·27 (3·17, 3·37)	1·45 (1·40, 1·50)	1·24 (1·17, 1·31)	0·001
RESPIRATORY	2·49 (2·40, 2·59)	1·44 (1·39, 1·51)	1·22 (1·15, 1·30)	0·001
MUSCULOSKELETAL	2·03 (1·97, 2·09)	1·28 (1·25, 1·32)	1·16 (1·12, 1·22)	0·001
ENDOCRINE/METABOLIC	2·02 (1·96, 2·09)	1·34 (1·30, 1·39)	1·16 (1·10, 1·22)	0·001
GENITOURINARY	1·83 (1·75, 1·92)	1·22 (1·17, 1·28)	1·13 (1·06, 1·21)	0·05
CIRCULATORY SYSTEM	1·74 (1·68, 1·81)	1·14 (1·09, 1·18)	1·09 (1·03, 1·16)	0·26
GASTROINTESTINAL	1·58 (1·53, 1·63)	1·15 (1·11, 1·18)	1·12 (1·07, 1·17)	0·33
SKIN	1·41 (1·34, 1·48)	1·13 (1·08, 1·18)	1·11 (1·03, 1·19)	0·16

eTable 9. Associations between ADHD and physical conditions across full-siblings, adjusting for ADHD status in co-sibling (outcome person)

**CIRCULATORY SYSTEM**	**OR (95% CIs)**
Hypertension	1·08 (1·03, 1·13)
Ischemic	1·16 (1·07, 1·25)
Pulmonary disease	1·02 (0·89, 1·16)
Atrial fibrallation	1·04 (0·97, 1·11)
Heart failure	1·14 (1·01, 1·28)
Stroke	1·16 (1·07, 1·27)
Peripheral vascular disease	1·11 (0·99, 1·24)
ENDOCRINE & METABOLIC DISEASE	
Type 1 diabetes mellitus	1·12 (1·04, 1·20)
Type 2 diabetes mellitus	1·17 (1·09, 1·26)
Thyroid	1·14 (1·08, 1·20)
Obesity	1·48 (1·41, 1·55)
Gout	1·27 (1·07, 1·50)
**GASTROINTESTINAL SYSTEM**	
Celiac disease	1·04 (0·92, 1·17)
Ulcer/chronic gastritis	1·25 (1·15, 1·35)
Acute appendicitis	1·05 (1·00, 1·10)
Fatty liver disease	1·24 (0·97, 1·59)
Alcohol-related liver disease	1·74 (1·41, 2·14)
IBD	1·03 (0·96, 1·11)
Gallstone disease	1·18 (1·13 1·24)
**GENITOURINARY SYSTEM**	
Glomerular disease	1·22 (1·08, 1·38)
Urolithiasis	1·11 (1·05, 1·18)
Kidney infections	1·28 (1·19, 1·37)
**MUSCULOSKELETAL SYSTEM**	
Rheumatoid arthritis	1·02 (0·91, 1·14)
Arthrosis	1·10 (1·05, 1·16)
Connective tissue disease	1·12 (1·03, 1·22)
Dorsalgia (back pain)	1·32 (1·28, 1·36)
**NERVOUS SYSTEM**	
Parkinson’s disease	1·03 (0·77, 1·37)
Dementia	0·99 (0·77, 1·26)
Epilepsy	1·26 (1·18, 1·35)
Migraine	1·30 (1·23, 1·38)
Sleep disorder	1·37 (1·30, 1·45)
**RESPIRATORY SYSTEM**	
Asthma	1·31 (1·25, 1·37)
Chronic obstructive pulmonary Disease	1·59 (1·46,1·74)
**SKIN**	
Eczema	1·00 (0·93, 1·08)
Psoriasis	1·20 (1·13 1·28)

Table 10. Associations between ADHD and physical conditions within-individuals and between full- and maternal half-siblings: Including only sibling pairs born within 10 years apart

	OR (95% CIs)		P value
	Full sibs (n=4,052,815)	Half sibs (n=420,650)	Full vs half sibs
Hypertension	1·07 (1·01, 1·12)	1·03 (0·93, 1·15)	0·60
Ischemic heart disease	1·12 (1·03, 1·23)	1·13 (0·93, 1·37)	0·94
Pulmonary disease	1·06 (0·92, 1·21)	1·02 (0·77, 1·34)	0·80
Atrial fibrillation	1·02 (0·95, 1·10)	1·04 (0·90, 1·20)	0·85
Heart failure	1·13 (0·99, 1·28)	1·39 (1·08, 1·79)	0·15
Stroke	1·17 (1·06, 1·29)	1·02 (0·82, 1·27)	0·27
Peripheral vascular disease	1·11 (0·98, 1·25)	1·18 (0·92 1·51)	0·66
Type 1 diabetes mellitus	1·16 (1·08, 1·25)	1·00 (0·86, 1·17)	0·09
Type 2 diabetes mellitus	1·20 (1·11, 1·30)	1·07 (0·92, 1·24)	0·19
Thyroid disorders	1·18 (1·12, 1·25)	1·11 (1·00, 1·23)	0·31
Obesity	1·62 (1·54, 1·70)	1·23 (1·14, 1·33)	<0·001*
Gout	1·22 (1·01, 1·48)	1·27 (0·89, 1·81)	0·86
**GASTROINTESTINAL**			
Celiac disease	1·10 (0·97, 1·24)	1·10 (0·87, 1·38)	0·99
Ulcer/chronic gastritis	1·27 (1·16, 1·39)	1·25 (1·04, 1·50)	0·86
Acute appendicitis	1·06 (1·01, 1·11)	1·11 (1·01, 1·21)	0·42
Fatty liver disease	1·36 (1·04, 1·77)	1·14(0·68, 1·90)	0·55
Alcohol-related liver disease	1·96 (1·56, 2·57)	1·56 (1·07, 2·29)	0·32
IBD	0·95 (0·95, 1·11)	1·02 (0·88, 1·18)	0·98
Gallstone disease	1·21 (1·16 1·28)	1·13 (1.04, 1.23)	0·17
**GENITOURINARY**			
Glomerular disease	1·28 (1·12, 1·46)	1·34 (1·07, 1·69)	0·71
Urolithiasis	1·14 (1·07, 1·21)	1·08 (0·96, 1·21)	0·62
Kidney infections	1·37 (1·27, 1·48)	1·16 (1·03, 1·32)	0·03
**MUSCULOSKELETAL**			
Rheumatoid arthritis	1·05 (0·93, 1·19)	1·03 (0·82, 1·31)	0·90
Arthrosis	1·10 (1·05, 1·16)	1·03 (0·92, 1·14)	0·24
Connective tissue disease	1·17 (1·07, 1·28)	1·14 (0·96, 1·36)	0·83
Dorsalgia (back pain)	1·40 (1·35, 1·45)	1·24 (1·17, 1·32)	0·002*
**NERVOUS SYSTEM**			
Parkinson's disease	1·09 (0·79, 1·52)	1·74 (0·93, 3·24)	0·20
Dementia	1·06 (0·80, 1·40)	1·31 (0·67, 2·57)	0·56
Epilepsy	1·41 (1·31, 1·51)	1·33 (1·17, 1·51)	0·41
Migraine	1·38 (1·30, 1·47)	1·11 (0·99, 1·25)	<0·001*
Sleep disorders	1·60 (1·52, 1·70)	1·26 (1·13, 1·41)	<0·001*
**RESPIRATORY**			
Asthma	1·44 (1·37, 1·50)	1·19 (1·09, 1·28)	<0·001*
COPD	1·67 (1·51,1·84)	1·32 (1·08, 1·63)	0·05
**SKIN**			
Eczema	1·02 (0·95, 1·09)	1·06 (0·93, 1·21)	0·58
Psoriasis	1·22 (1·14, 1·31)	1·01 (0·98, 1·25)	0·17

OR: Odds ratios, CIs: Confidence intervals, IBD: Irritable bowel syndrome, COPD: chronic obstructive pulmonary disease. Analyses are adjusted for sex and birthyear. *p-value significance threshold <0.007, FDR corrected).

eTable 11. Prevalence of conditions and associations between ADHD and physical conditions within-individuals and between full- and maternal half-siblings: in males (N = 2,449,146)

	N (%)	OR (95% CIs)		
		Within ind.	Full sibs	Half sibs
**ADHD**	34,823 (1·42)			
**CIRCULATORY SYSTEM**				
Hypertension	254,871 (10·41)	1·71 (1·60, 1·84)	1·14 (1·07, 1·21)	1·04 (0·94, 1·16)
Ischemic heart disease	124,967 (5·10)	1·38 (1·23, 1·56)	1·18 (1·07, 1·29)	1·16 (0·98, 1·37)
Pulmonary disease	19,724 (0·81)	1·83 (1·51, 2·22)	1·06 (0·88, 1·27)	1·14 (0·83, 1·56)
Atrial fibrillation	105,599 (4·31)	1·21 (1·09, 1·34)	1·07 (0·98, 1·17)	1·07 (0·92, 1·23)
Heart failure	46,928 (1·92)	1·65 (1·40, 1·94)	1·17 (1·00, 1·35)	1·18 (0·91, 1·52)
Stroke	58,946 (2·41)	1·81 (1·59, 2·06)	1·15 (1·02, 1·29)	1·20 (0·98, 1·46)
Peripheral vascular disease	38,005 (1·55)	1·86 (1·57, 2·21)	1·03 (0·88, 1·20)	1·13 (0·87 1·46)
**ENDOCRINE/METABOLIC**				
Type 1 diabetes mellitus	47,560 (1·94)	1·54 (1·39, 1·70)	1·17 (1·07, 1·28)	0·99 (0·84, 1·17)
Type 2 diabetes mellitus	95,158 (3·89)	1·90 (1·73, 2·10)	1·23 (1·12, 1·34)	0·95 (0·81, 1·11)
Thyroid disorder	25,168 (1·03)	2·00 (1·77, 2·26)	1·18 (1·04, 1·34)	1·02 (0·81, 1·29)
Obesity	36,636 (1·50)	2·89 (2·68, 3·13)	1·62 (1·48, 1·77)	1·33 (1·18, 1·51)
Gout	16,339 (0·67)	1·70 (1·39, 2·08)	1·33 (1·10, 1·60)	1·16 (0·85, 1·58)
**GASTROINTESTINAL**				
Celiac disease	6,368 (0·26)	1·24 (1·01, 1·54)	1·05 (0·85, 1·29)	1·00 (0·69, 1·45)
Ulcer/chronic gastritis	40,888 (1·67)	2·31 (2·06, 2·59)	1·32 (1·18, 1·47)	1·43 (1·19, 1·73)
Acute appendicitis	86,400 (3·53)	1·19 (1·11, 1·28)	1·07 (1·00, 1·14)	1·07 (0·96, 1·18)
Fatty liver disease	3,283 (0·13)	2·66 (2·04, 3·48)	1·48 (1·09, 2·01)	1·07 (0·59, 1·94)
Alcohol-related liver disease	7,421 (0·30)	4·87 (4·00, 5·91)	1·89 (1·48, 2·42)	1·24 (0·84, 1·82)
IBD	32,450 (1·32)	1·19 (1·06, 1·34)	1·01 (0·91, 1·13)	1·12 (0·96, 1·31)
Gallstone disease	63,993 (2·61)	1·88 (1·72, 2·06)	1·29 (1·18 1·40)	1·03 (0·89, 1·19)
**GENITOURINARY**				
Glomerular disease	13,668 (0·56)	1·91 (1·60, 2·29)	1·22 (1·03, 1·44)	1·29 (1·01, 1·64)
Urolithiasis	81,392 (3·32)	1·42 (1·30, 1·55)	1·07 (0·99, 1·16)	1·02 (0·90, 1·16)
Kidney infections	17,963 (0·73)	2·16 (1·82, 2·56)	1·40 (1·20, 1·64)	1·22 (0·92, 1·60)
**MUSCULOSKELETAL**				
Rheumatoid arthritis	12,629 (0·52)	1·05 (0·80, 1·39)	0·82 (0·64, 1·05)	1·14 (0·80, 1·62)
Arthrosis	155,406 (6·35)	1·26 (1·17, 1·36)	1·07 (1·00, 1·14)	1·12 (1·00, 1·25)
Connective tissue disease	26,099 (1·07)	1·58 (1·33, 1·87)	1·10 (0·94, 1·27)	1·10 (0·85, 1·41)
Dorsalgia (back pain)	140,854 (5·75)	2·38 (2·27, 2·49)	1·42 (1·34, 1·48)	1·23 (1·14, 1·32)
**NERVOUS SYSTEM**				
Parkinson's disease	7,619 (0·31)	1·35 (0·88, 2·07)	0·91 (0·63, 1·33)	2·27 (1·17, 4·41)
Dementia	10,618 (0·43)	2·71 (1·92, 3·82)	1·07 (0·77, 1·50)	1·01 (0·45, 2·27)
Epilepsy	33,804 (1·38)	3·24 (3·00, 3·49)	1·37 (1·25, 1·51)	1·16 (1·01, 1·35)
Migraine	18,170 (0·74)	1·92 (1·71, 2·15)	1·57 (1·41, 1·75)	1·18 (0·98, 1·42)
Sleep disorder	86,059 (3·51)	3·83 (3·63, 4·04)	1·51 (1·42, 1·62)	1·33 (1·20, 1·48)
**RESPIRATORY**				
Asthma	58,106 (2·37)	2·15 (2·01, 2·28)	1·34 (1·25, 1·44)	1·21 (1·09, 1·35)
COPD	34,998 (1·43)	3·15 (2·75, 3·61)	1·76 (1·55, 2·00)	1·23 (0·97, 1·57)
**SKIN**				
Eczema	16,510 (0·67)	1·41 (1·23, 1·60)	0·99 (0·87, 1·12)	0·93 (0·75, 1·14)
Psoriasis	42,899 (1·75)	1·33 (1·20, 1·47)	1·24 (1·13, 1·36)	1·09 (0·94, 1·26)

eTable 12. Prevalence of conditions and associations between ADHD and physical conditions within-individuals and between full- and maternal half-siblings: in females (N = 2,340,653)

	N (%)	OR (95% CIs)		
		Within ind.	Full sibs	Half sibs
**ADHD**	27,137 (1·16)			
**CIRCULATORY SYSTEM**				
Hypertension	207,242 (8·85)	1·47 (1·44, 1·71)	1·05 (0·98, 1·13)	1·03 (0·92, 1·17)
Ischemic heart disease	59,770 (2·55)	1·25 (1·03, 1·51)	1·14 (1·00, 1·30)	1·04 (0·82, 1·32)
Pulmonary disease	18,393 (0·79)	2·02 (1·67, 2·44)	1·04 (0·87, 1·25)	1·01 (0·76, 1·34)
Atrial fibrillation	63,387 (2·71)	1·55 (1·37, 1·75)	1·02 (0·92, 1·14)	1·06 (0·90, 1·24)
Heart failure	24,143 (1·03)	1·81 (1·44, 2·27)	1·14 (0·93, 1·38)	1·19 (0·86, 1·64)
Stroke	38,444 (1·64)	2·08 (1·77, 2·43)	1·25 (1·09, 1·43)	0·87 (0·68, 1·12)
Peripheral vascular disease	24,181 (1·03)	1·73 (1·44, 2·09)	1·26 (1·08, 1·48)	1·10 (0·85 1·43)
**ENDOCRINE/METABOLIC**				
Type 1 diabetes mellitus	33,181 (1·42)	1·51 (1·33, 1·70)	1·13 (1·01, 1·26)	0·84 (0·70, 1·01)
Type 2 diabetes mellitus	59,051 (2·52)	2·23 (1·99, 2·50)	1·18 (1·06, 1·32)	1·16 (0·98, 1·37)
Thyroid disorder	124,693 (5·33)	1·66 (1·56, 1·77)	1·18 (1·12, 1·25)	1·13 (1·03, 1·24)
Obesity	77,132 (3·30)	2·67 (2·53, 2·82)	1·60 (1·52, 1·69)	1·24 (1·14, 1·33)
Gout	4,347 (0·19)	2·19 (1·42, 3·37)	1·26 (0·85, 1·76)	0·74 (0·35, 1·55)
**GASTROINTESTINAL**				
Celiac disease	12,584 (0·54)	1·47 (1·26, 1·72)	1·08 (0·94, 1·25)	0·92 (0·73, 1·17)
Ulcer/chronic gastritis	32,328 (1·38)	2·68 (2·38, 3·02)	1·30 (1·15, 1·47)	1·26 (1·01, 1·48)
Acute appendicitis	68,172 (2·91)	1·31 (1·21, 1·43)	1·06 (0·99, 1·14)	1·06 (0·95, 1·18)
Fatty liver disease	2,542 (0·11)	3·38 (2·39, 4·79)	1·19 (0·79, 1·79)	0·93 (0·50, 1·74)
Alcohol-related liver disease	2,927 (0·13)	4·22 (2·91, 6·13)	1·92 (1·29, 2·87)	1·98 (1·27, 3·07)
IBD	32,340 (1·38)	1·28 (1·13, 1·44)	1·08 (0·97, 1·20)	1·00 (0·85, 1·17)
Gallstone disease	149,538 (6·39)	1·76 (1·66, 1·86)	1·19 (1·13 1·26)	1·18 (1·09, 1·27)
**GENITOURINARY**				
Glomerular disease	9,298 (0·40)	2·24 (1·88, 2·66)	1·36 (1·14, 1·62)	1·13 (0·85, 1·50)
Urolithiasis	47,190 (2·02)	1·79 (1·63, 1·97)	1·22 (1·12, 1·33)	1·16 (1·03, 1·31)
Kidney infections	43,332 (1·85)	2·45 (2·27, 2·64)	1·35 (1·25, 1·46)	1·11 (0·99, 1·24)
**MUSCULOSKELETAL**				
Rheumatoid arthritis	30,356 (1·30)	1·10 (0·91, 1·31)	1·09 (0·96, 1·24)	1·06 (0·87, 1·31)
Arthrosis	167,011 (7·14)	1·34 (1·23, 1·46)	1·17 (1·09, 1·25)	0·87 (0·77, 0·98)
Connective tissue disease	42,766 (1·83)	1·89 (1·69, 2·12)	1·20 (1·08, 1·32)	1·09 (0·92, 1·28)
Dorsalgia (back pain)	157,222 (6·72)	2·41 (2·31, 2·53)	1·39 (1·33, 1·46)	1·23 (1·15, 1·32)
**NERVOUS SYSTEM**				
Parkinson's disease	4,950 (0·21)	1·78 (1·06, 3·00)	1·22 (0·79, 1·89)	1·13 (0·55, 2·34)
Dementia	10,111 (0·43)	2·07 (1·13, 3·22)	0·94 (0·65, 1·36)	1·77 (0·95, 3·28)
Epilepsy	28,338 (1·21)	2·72 (2·49, 2·97)	1·43 (1·30, 1·57)	1·25 (1·09, 1·44)
Migraine	52,256 (2·23)	2·00 (1·86, 2·15)	1·31 (1·22, 1·40)	1·11 (1·00, 1·23)
Sleep disorder	36,921 (1·58)	6·70 (6·28, 7·15)	1·73 (1·58, 1·89)	1·32 (1·15, 1·52)
**RESPIRATORY**				
Asthma	85,722 (3·66)	2·67 (2·53, 2·81)	1·46 (1·38, 1·54)	1·22 (1·12, 1·32)
COPD	39,870 (1·70)	3·37 (2·97, 3·83)	1·62 (1·43, 1·83)	1·39 (1·15, 1·68)
**SKIN**				
Eczema	33,547 (1·43)	1·44 (1·31, 1·58)	1·05 (0·97, 1·14)	1·10 (0·97, 1·24)
Psoriasis	46,740 (2·00)	1·46 (1·32, 1·61)	1·21 (1·11, 1·32)	1·23 (1·08, 1·40)

eTable 13. Univariate ACE models with bootstrap 95% confidence intervals

Disorder	A	95% CI: Lower, upper	C	95% CI: Lower, upper	E	95% CI: Lower, upper
ADHD	0·59	0·52, 0·66	0·05	0·02, 0·08	0·36	0·32, 0·41
Sleep disorder	0·24	0·16, 0·28	0·03	0·01, 0·07	0·73	0·71, 0,78
Migraine	0·19	0·09, 0·25	0·04	0·01, 0·09	0·77	0·74, 0·83
Asthma	0·34	0·28, 0·38	0·02	0·00, 0·05	0·64	0·62, 0·67
COPD	0·35	0·21, 0·38	0·02	0·00, 0·08	0·64	0·62, 0·71
Dorsalgia	0·17	0·12, 0·21	0·02	0·01, 0·05	0·80	0·78, 0·83
Kidney*	0·13	0·03, 0·15	0·00	0·00, 0·05	0·87	0·85, 0·93
Obesity	0·42	0·35, 0·48	0·07	0·04, 0·10	0·52	0·48, 0·55
Type 1 diabetes mellitus	0·48	0·37, 0·52	0·02	0·01, 0·08	0·50	0·47, 0·56
**NERVOUS SYSTEM**	0·15	0·10, 0·18	0·02	0·01, 0·04	0·83	0·82, 0·86
**METABOLIC**	0·35	0·31, 0·37	0·02	0·01, 0·04	0·64	0·62, 0·66
**RESPIRATORY**	0·35	0·28, 0·36	0·01	0·00, 0·04	0·65	0·64, 0·68
**MUSCULOSKELETAL**	0·20	0·15, 0·21	0·01	0·01, 0·03	0·79	0·78, 0·81

*For kidney disease, the univariate bootstrapped 95% CIs were based on 300 bootstrap samples, due to numerical instability (we resampled within these 300 to get a total of 1000 bootstrap samples).
A: Additive genetic component, C: Shared environment component, E: Non-shared environment component.

eTable 14. A, C and E correlations with bootstrap 95% confidence intervals

Disorder	r_{ph}	95% CI: Lower, upper	r_A	95% CI: Lower, upper	r_C	95% CI: Lower, upper	r_E	95% CI: Lower, upper
Sleep disorder	0·28	0·27, 0·28	0·21	0·10, 0·41	0·99	0·12, 1·00	0·32	0·24, 0·36
Migraine	0·11	0·10, 0·12	0·31	0·08, 0·60	-0·06	-1·00, 1·00	0·02	-0·06, 0·10
Asthma	0·15	0·15, 0·16	0·15	0·08, 0·28	0·76	-0·09, 1·00	0·13	0·07, 0·17
COPD	0·18	0·16, 0·19	0·24	0·10, 0·56	0·72	-1·00, 1·00	0·11	-0·04, 0·18
Dorsalgia	0·17	0·16, 0·17	0·24	0·14, 0·40	0·73	-0·01, 1·00	0·13	0·07, 0·16
Kidney	0·13	0·12, 0·15	0·37	0·12, 1·00	-0·99	-1·00, 1·00	0·06	-0·001, 0·14
Obesity	0·17	0·16, 0·18	0·20	0·09, 0·33	0·45	-0·03, 1·00	0·10	0·01, 0·18
Type 1 diabetes mellitus	0·06	0·05, 0·08	0·20	0·09, 0·37	-0·99	-1·00, -0·20	-0·02	-0·11, 0·06
**NERVOUS SYSTEM**	0·23	0·23, 0·24	0·22	0·16, 0·44	0·99	0·17, 1·00	0·25	0·19, 0·27
**METABOLIC**	0·14	0·13, 0·15	0·19	0·08, 0·30	0·42	-0·34, 1·00	0·09	0·03, 0·15
**RESPIRATORY**	0·16	0·16, 0·17	0·22	0·11, 0·33	0·99	-0·13, 1·00	0·10	0·05, 0·17
**MUSCULOSKEL**	0·14	0·14, 0·15	0·29	0·12, 0·38	0·38	-0·28, 1·00	0·07	0·04, 0·14

r_{ph}: phenotypic correlation, r_A: additive genetic correlation, r_C: shared environment correlation, r_E: non-shared environment correlation.

eTable 15. Explained variance from A, C and E with bootstrap 95% confidence intervals

Disorder	biv_A	Lower, upper	biv_C	Lower, upper	biv_E	Lower, upper
Sleep disorder	0·28	0·11, 0·54	0·12	0·02, 0·20	0·60	0·44, 0·70
Migraine	0·95	0·24, 1·62	-0·02	-0·32, 0·32	0·07	-0·30, 0·49
Asthma	0·44	0·21, 0·81	0·15	-0·01, 0·26	0·41	0·20, 0·54
COPD	0·61	0·17, 1·29	0·11	-0·19, 0·32	0·28	-0·11, 0·53
Dorsalgia	0·46	0·25, 0·77	0·14	0·00, 0·23	0·40	0·22, 0·53
Kidney	0·77	0·22, 1·24	-0·03	-0·24, 0·21	0·26	-0·01, 0·59
Obesity	0·60	0·25, 0·97	0·14	-0·01, 0·31	0·25	0·03, 0·47
Type 1 diabetes mellitus	1·67	0·74, 2·79	-0·53	-1·04, -0·10	-0·14	-0·84, 0·36
**NERVOUS SYSTEM**	0·28	0·18, 0·52	0·13	0·02, 0·18	0·59	0·44, 0·65
**METABOLIC**	0·62	0·26, 0·95	0·08	-0·07, 0·23	0·30	0·09, 0·52
**RESPIRATORY**	0·60	0·27, 0·87	0·09	-0·02, 0·22	0·31	0·14, 0·52
**MUSCULOSKEL**	0·69	0·27, 0·88	0·06	-0·05, 0·22	0·25	0·16, 0·52
